# A guide to measuring phagosomal dynamics

**DOI:** 10.1111/febs.15506

**Published:** 2020-08-16

**Authors:** Roni Levin‐Konigsberg, Adriana R. Mantegazza

**Affiliations:** ^1^ Department of Genetics Stanford University School of Medicine Stanford CA USA; ^2^ Department of Pathology and Laboratory Medicine Children's Hospital of Philadelphia Philadelphia PA USA; ^3^ Department of Pathology and Laboratory Medicine Perelman School of Medicine University of Pennsylvania Philadelphia PA USA

**Keywords:** antigen presentation, autophagy, dendritic cell, inflammation, macrophage, membrane contact sites, pattern recognition receptors, phagocytosis, phagosome maturation, phagosome resolution

## Abstract

Phagocytosis is an essential mechanism for immunity and homeostasis, performed by a subset of cells known as phagocytes. Upon target engulfment, *de novo* formation of specialized compartments termed phagosomes takes place. Phagosomes then undergo a series of fusion and fission events as they interact with the endolysosomal system and other organelles, in a dynamic process known as phagosome maturation. Because phagocytes play a key role in tissue patrolling and immune surveillance, phagosome maturation is associated with signaling pathways that link phagocytosis to antigen presentation and the development of adaptive immune responses. In addition, and depending on the nature of the cargo, phagosome integrity may be compromised, triggering additional cellular mechanisms including inflammation and autophagy. Upon completion of maturation, phagosomes enter a recently described phase: phagosome resolution, where catabolites from degraded cargo are metabolized, phagosomes are resorbed, and vesicles of phagosomal origin are recycled. Finally, phagocytes return to homeostasis and become ready for a new round of phagocytosis. Altogether, phagosome maturation and resolution encompass a series of dynamic events and organelle crosstalk that can be measured by biochemical, imaging, photoluminescence, cytometric, and immune‐based assays that will be described in this guide.

## Graphical abstract

After cargo engulfment by phagocytic cells, phagosomes sequentially undergo maturation and resolution in a labyrinth journey that involves crosstalk with the endolysosomal system, as well as other organelles and cellular pathways. In this guide, we discuss different methodologies to study this dynamic process, highlighting their advantages and limitations while stressing areas of the field in need of new strategies to overcome technical challenges and address gaps in our current knowledge.

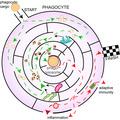

**Abbreviations**
DC: Dendritic cell
EEA1: Early endosome antigen 1
ELISA: Enzyme‐linked immunosorbent assay
EM: Electron microscopy
ER: Endoplasmic reticulum
ERC: Endosomal recycling compartment
ERGIC: ER–Golgi intermediate compartment
ESCRT: Endosomal sorting complexes required for transport
Fc: Fragment crystallizable
FRET: Fluorescence resonance energy transfer
H2: histocompatibility 2
HLA: human leukocyte antigen
IF: Immunofluorescence
IgG: Immunoglobulin G
IL‐12: Interleukin‐12
IL‐1β: Interleukin‐1 beta
IL‐2: Interleukin‐2
IL‐6: Interleukin‐6
LAMP: Lysosome‐associated membrane protein
LLSM: Lattice light‐sheet microscopy
MCS: Membrane contact sites
MFI: Mean fluorescence intensity
MHC: Major histocompatibility complex
mTORC1: Mechanistic target of rapamycin complex 1
NADPH: Nicotinamide adenine dinucleotide phosphate
NOX2: NADPH oxidase 2
OVA: Ovalbumin
pH: Potential for hydrogen
PRR: Pattern recognition receptor
RBC: Red blood cells
ROS: Reactive oxygen species
TCR: T‐cell receptor
TGN: Trans‐Golgi Network
TLR: Toll‐like receptor

## Introduction

Initially described by Elie Metchnikoff more than 100 years ago [1, 2], phagocytosis is conventionally defined as the regulated uptake of large particulate matter by specialized cells into membrane‐bound vacuoles termed phagosomes. Indeed, phagocytic cells (phagocytes) recognize and internalize a wide array of targets for diverse purposes. Hence, ‘phagocytosis’ serves as an umbrella term that encompasses all modalities of particle internalization, and while phagocytes spend a significant amount of energy and resources to engulf particles, the definition neglects the ultimate goal of the phenomenon: processing the captured prey. In addition, in immunity, phagocytosis is not a silent process; the prey is not kept secret. On the contrary, in multicellular organisms, phagosomes become sensing and signaling hubs that communicate with other organelles and signaling pathways to optimize the phagocytic process and mount the right type of immune response [3, 4, 5]. Regardless of whether phagocytosis is used as a means for nutrient acquisition—as is the case with some unicellular organisms—to eliminate imminent threats or for homeostatic purposes in metazoans, internalized matter must be degraded, the resulting catabolites must be resolved, and the information gathered along the way must be conveyed throughout the cell.

**Fig. 1:**
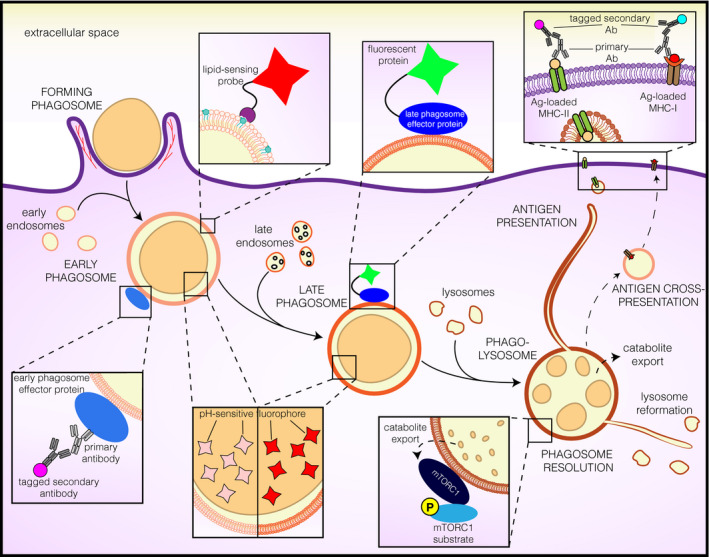
The life cycle of phagosomes. Phagosomes are formed at the plasmalemmal level in an actin‐dependent manner. Once inside cells, phagosomes mature through sequential fusion with early endosomes, late endosomes, and lysosomes. During maturation, phagosomes acquire effector proteins that can be probed in cells by immunolabeling and genetically encoded protein chimeras. Additionally, the lipid composition of the phagosomal membrane changes throughout maturation; such changes can be measured using genetically encoded lipid‐sensing probes. In some phagocytes, as phagosomes mature, their lumen acidifies; this can be assessed through the use of pH‐sensitive fluorophores. In some phagocytes, such as DCs, phagosomes are autonomous signaling organelles equipped for cargo degradation, peptide loading, and antigen presentation. The latter can be measured when peptides are loaded intracellularly or presented at the plasma membrane by immunolabeling methods or by measuring subsequent T‐cell activation. Finally, after cargo degradation, phagosomes undergo resolution, which entails catabolite export, compartment resorption, and lysosome reformation. Export of catabolites (such as specific amino acids) can activate mTORC1 in the cytosolic leaflet of lysosomal membranes. mTORC1 activation can be measured by changes (e.g., phosphorylation) in its substrates.

**Fig. 2:**
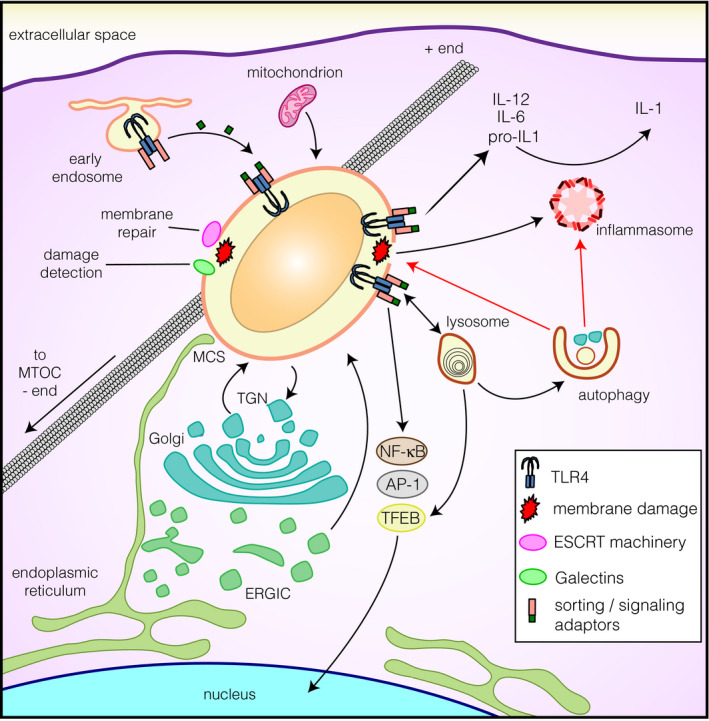
Signaling and crosstalk of the maturing phagosome. During maturation, phagosomes are linked to microtubules through which they are transported centripetally toward the microtubule‐organizing center. Additionally, phagosomes interact with the ER, the ERGIC, the TGN and mitochondria. As phagosomes mature, they become autonomous signaling entities by acquiring PRRs from endosomal compartments (e.g., TLR4). Receptor signaling promotes the production of proinflammatory cytokines—through NF‐kB and AP‐1 transcription factor activation—and enhances cargo degradation and antigen presentation depicted in Fig. 1. Some receptors also induce TFEB/TFE3 translocation to the nucleus and subsequent transcription of the coordinated lysosomal expression and regulation and proinflammatory gene networks. Additionally, some phagocytic cargoes can induce phagosomal membrane damage, which in turn results in phagosomal content leakage and inflammasome activation. These events can be targeted by autophagy. Additionally, mechanisms to detect and repair membrane damage include galectins and the ESCRT machinery, respectively, and can also be probed by immunoassays.

Because of this, the phagocytic process has been broadly divided into major mechanistic stages: phagosome formation, phagosome maturation, and, more recently, phagosome resolution [6]. While phagosome formation is a remarkable process whereby phagocytes recognize prey and internalize it by remodeling their plasma membrane and rearranging their actin cytoskeleton, and has been extensively reviewed [7, 8, 9], this guide aims to discuss methods to study the subsequent stages, namely phagosome maturation and phagosome resolution (Fig. 1). Phagosome maturation was initially defined as the fusion of phagosomes with lysosomes—or granules in neutrophils—for cargo degradation [10, 11]. Studies over the past 30 years have shown the complex molecular mechanisms that are necessary for a newly formed phagosome to be transformed into a degradative organelle—known as the phagolysosome—as it traffics and signals through the endocytic pathway (reviewed in Ref. [6, 12, 13, 14, 15, 16]. While it is widely known that phagosomes undergo a sequential series of fusion and fission events with endosomes and lysosomes [17, 18] (Fig. 1), it has become increasingly apparent that phagosomes also interact with several other organelles such as the endoplasmic reticulum (ER) [19], mitochondria [20, 21], and the trans‐Golgi network (TGN) [22] (Fig. 2). Moreover, only recently have several groups started investigating the consequences of breaching phagosome membrane integrity and the fate of the compartment and its catabolites following cargo degradation ([23, 24, 25]; Fig. 2). The importance of these phenomena is emphasized by fundamental processes such as inflammasome activation, induction of autophagy, antigen presentation, the recycling of organelles of the endocytic pathway, and the return to homeostasis so that the cells become readily available for additional rounds of phagocytosis. These late events—catabolite management and compartment resorption—encompass the recently described phase termed phagosome resolution [6]. The spatial and temporal sequence that orchestrates the mechanisms of phagosome maturation and resolution is tightly coordinated by a plethora of molecular events that can be studied by diverse methods. Here, we provide a guideline for diverse methods to study changes in phagosomal composition, signaling and integrity, cargo degradation, and phagosome resolution.

## Chapter 1. Traditional methods to assess phagosome maturation

### Changes in the biochemical composition and properties of the maturing phagosome

#### At a glance

As soon as a nascent phagosome is formed, it undergoes a drastic biochemical transformation, altering the composition of both its lumen and limiting membrane. The goal of these changes is to transform an ‘innocuous’ environment that resembles the extracellular milieu, into a hostile one that favors lytic reactions. This transition is driven by the trafficking and maturation of phagosomes through the endocytic pathway. Maturation is characterized by fusion and fission events with early and late endosomes, post‐Golgi vesicles, and lysosomes [17, 18], exquisitely coupled to the delivery and activation of a hydrolytic, oxidative, and acidifying machinery, as well as by centripetal movement of the vacuole along microtubules [26, 27] and binding to F‐actin [28, 29]. Throughout maturation, the phagosome dynamically acquires stage‐specific proteins and lipids (reviewed in Ref. [6, 13, 14, 15, 30, 31]. These molecules can then be used as proxies to study the state of maturation of phagosomes by the methods described below. Perhaps, one of the most remarkable aspects of phagosome maturation is that the lumen of phagosomes—with some exceptions depending on the type of phagocyte [32, 33]—undergoes acidification. This Potential for hydrogen (pH) decrease is in principle necessary for complete degradation of phagosomal cargo, since several hydrolases acquired by phagolysosomes have acidic pH optima. Additionally, reactive oxygen species (ROS) are generated in phagosomes to various degrees and with temporal variations depending on the nature of the phagocyte [32, 34, 35].

### Biochemical methods

#### Phagosome isolation for biochemical studies

The principle of the method is the separation of phagosomes from cell homogenates based on their differential densities, as phagocytic cargoes are the predominant determinant of the density of the compartment. Because they have a density that is markedly different from that of cellular organelles, latex beads have been widely used as model phagocytic targets. These methods, originally developed in the late 1960s and early 1970s [36, 37], can be performed with a one‐step density gradient [38]. Notably, phagosome isolation methodologies have been further optimized in order to increase homogeneity and purity [39, 40, 41, 42]. Isolation techniques of physiologically relevant phagocytic prey such as bacteria, albeit significantly more challenging, have also been developed [43, 44, 45, 46, 47, 48].

Many purification methods are based on Percoll or sucrose density gradients [46, 49]. Phagocytes in culture are initially challenged with the phagocytic prey of choice and incubated for the desired time. Then, cells are scraped off or otherwise lifted and homogenized in the presence of protease inhibitors. The homogenate is then layered over a Percoll gradient or fractionated on a discontinuous sucrose gradient and centrifuged. Phagosomes are then collected from the appropriate interphase and their purity and properties assessed through downstream methods. Alternative strategies for phagosome isolation have emerged more recently, such as magnetic separation strategies, in which phagocytic targets can either be intrinsically magnetic (magnetic beads) or prelabeled with magnetic nanoparticles [44, 47, 50, 51, 52].

#### Downstream analysis methods

##### Proteomics

Some of the first systematic biochemical determinations of isolated phagosomes were done through two‐dimensional gel electrophoresis using radiolabeled metabolites [38]. These studies rely on the identification of proteins by comigration with a limited number of standard proteins from a database. Phagosomes can also be analyzed by immunoblotting, whereby enrichment of specific proteins during the progression of phagocytosis can be assessed.

The development of advanced proteomic techniques, specifically high‐performance liquid chromatography coupled to mass spectrometry, allowed large‐scale, comprehensive analysis of the composition of phagosomes. Indeed, the evolving nature of phagosomes and the relative ease to isolate them from cell homogenates make phagocytosis an attractive process for proteomic studies. During the past ~ 20 years, diverse studies have systematically defined the phagosomal proteome at different stages of the process [39, 53, 54]. One of the major advantages of this technique is the possibility of performing unbiased experiments throughout the various stages of phagocytosis. Indeed, the development of quantitative proteomic techniques has shed light on the dynamics and composition of maturing phagosomes. In order for these approaches to represent individual stages of phagocytosis, it is highly recommended to synchronize the internalization through pulse‐chase procedures (i.e., allow the phagocytes to engage prey for a limited period of time, before thoroughly washing unbound targets). However, as a bulk, ‘end‐point’ method, proteomic analysis of a population does not provide information of individual phagosomes and it lacks spatial resolution. Perhaps, the major caveat of this approach is that complete purification of phagosomes is nearly impossible to achieve due to the complex interactions of the compartment with virtually every organelle in the cytoplasm. At the same time, this methodology allows to uncover possible membrane crosstalk between phagosomes and other organelles. Thus, criteria to discern between contaminants and actual organelle interactions are essential to control these approaches. This can be accomplished by incubating cargo with lysed cells, as one way to detect nonspecific interactions. Notably, over the years major strides have been made to maximize the purity of isolated phagosomes [39, 40, 42, 55, 56].

It is important to stress that the use of mass spectrometry as a platform to examine phagosomes carries the potential to fill important gaps in the field. While the phagosomal proteome has been widely investigated, other phagosomal ‘omics’ such as the lipidome and glycome have been studied to a significantly lesser extent [57, 58]. Because of the increasingly recognized contribution of lipids, carbohydrates and metabolites to phagosomal dynamics, defining changes in their molecular landscapes in phagosomes, will likely render important advances in the field, especially in the understanding of host–pathogen interactions [59].

##### Cell‐free fusion

Although exquisitely coordinated in time and space, each stage of phagocytosis is remarkably complex. Thus, accurately dissecting mechanisms at a molecular level can be challenging when studying phagocytosis in intact cells. To circumvent this, several groups have developed *in vitro* cell‐free systems, which have been particularly useful to study phagosome maturation [17, 60, 61, 62, 63]. While these methods still rely on the use of phagosomes isolated from phagocytes, they are unique in that they utilize *in vitro* incubation of phagosomes with isolated cellular components. For these experiments, endocytic components of phagocytic cells are isolated and then incubated with purified phagosomes in the presence of a cytosolic extract from phagocytes with added adenosine triphosphate and protease inhibitors. This setup enables a stringent temporal control of phagocytic maturation events. After incubation, phagosomes can be analyzed by immunoblotting and electron and fluorescence microscopy, depending on the original experimental design. Cell‐free systems were more commonly used with latex bead‐containing phagosomes, though more recent efforts have allowed to study bacterium‐containing vacuoles [62]. The versatility of these techniques was emphasized by studies showing binding of phagosomes to microtubules [64] and F‐actin [28] as well as *de novo* F‐actin assembly at phagosomal membranes [65]. However, caution should be used while designing this type of experiments, given that many phagocytic events are multifactorial and require several components and organelles.

### Imaging and fluorescence‐based methods

Because of the highly localized, dynamic and transient nature of phagosome maturation, microscopy techniques that provide high spatial and temporal resolution have enabled significant advances in the field. More recently, the continuous improvements in the resolution of imaging techniques have allowed for the identification of subphagosomal structures and regions [66]. Additionally, specialized probes allow for qualitative and quantitative determination of phagosomal pH and ROS. Finally, fluorescence‐based methods can also be used for bulk/ high‐throughput measurements of phagosomes and phagocytes, and can also be adapted to flow cytometry. These methods will be described below.

#### Immunolabeling

Immunolabeling methods are useful techniques to probe protein recruitment to phagosomes. After challenging phagocytes with phagocytic targets, cells can be fixed at specific times during the process. Cells are then permeabilized and probed with antibodies raised against the proteins of interest. After washing, samples are probed with a tagged secondary antibody [e.g., with fluorophores for immunofluorescence (IF) microscopy or flow cytometry, with gold particles for electron microscopy (EM)]. As in most maturation assessments, it is critical to synchronize phagocytosis through pulse‐chase protocols. Additionally, specific stage markers [i.e., early endosome antigen 1 (EEA1) for the early maturation stages; lysosome‐associated membrane proteins (LAMP) for late maturation stages; or prelabeling lysosomes with fixable dextrans using pulse‐chase protocols; Table 1] can be probed in parallel to confirm the maturation state of individual phagosomes. One of the main advantages of traditional imaging techniques is that they are compatible with primary cells and immortalized cell lines. Additionally, they represent the best option to study the localization of endogenous proteins within cells. However, it is worth noting that reliable antibodies are not always available for proteins of interest. Moreover, the number of proteins that can be probed per experiment is limited either by the species in which antibodies are raised (only one species per antibody), by the number of available fluorophores or number of fluorescent channels in the microscopy setup (for IF), or by the different sizes of gold particles (for EM). Thus, most experimental setups can probe 2–3 proteins. Additionally, the cell fixation and permeabilization methods that yield optimal results vary between antibodies and should be optimized.

**Table 1:** Selected reagents used in phagocytosis and phagosome maturation and crosstalk assays.

Reagent	Description	Applications	References
General phagocytosis assays			
Phagocytic targets			
Polystyrene beads	Inert particles	IF; immunoblot; proteomics; live‐cell imaging	[42, 96, 176]
Magnetic particles	Inert particles	Magnetic isolation of phagosomes and phagosome‐containing cells	[51, 177]
RBC	Cell targets	Imaging‐based methods; shear stress assessment	[166, 178]
Apoptotic cells	Cell targets	Imaging	[179, 180]
*E. coli*–OVA	Model Ag‐expressing bacteria	Immunoassays	[126]
STm‐OVA	Model Ag‐expressing bacteria	Immunoassays	[128]
*L. monocytogenes*–OVA	Model Ag‐expressing bacteria	Immunoassays	[181]
Zymosan	Yeast cell wall glycan component	Imaging‐based methods	[177, 182]
Immunoglobulin G (IgG)‐opsonized particles	Polystyrene bead opsonization (for Fc receptor‐mediated phagocytosis)	lF; live‐cell imaging	[176]
IgG‐opsonized RBC	RBC opsonization (for Fc receptor‐mediated phagocytosis)	Imaging‐based methods	[177, 178]
Phagosome maturation assays			
Proteolytic activity			
OVA degradation	Quantitative assessment	Flow cytometry	[67]
DQ‐BSA	Qualitative assessment	Fluorescence microscopy	[183, 184, 185]
pH measurement			
phRodo dyes	Qualitative assessment of acidification	Fluorescence microscopy	[186]
Cresyl violet	Qualitative assessment of acidification	Fluorescence microscopy	[187]
Fluorescein isothiocyanate (FITC)	Quantitative assessment	Ratiometric imaging	[33]
FITC/AF647	Quantitative assessment	Ratiometric flow cytometry	[67]
Oregon green 488 succinimidyl ester	Quantitative assessment	Ratiometric imaging	[88]
ROS measurement			
Luminol	Peroxidase‐dependent detection of O_2_^−^	Luminometry	[80]
p‐Hydroxyphenylacetate	Peroxidase‐dependent detection of H_2_O_2_	Fluorometry	[80]
Dihydrorhodamine 123	General detection of ROS by oxidation of fluorophore	Fluorometry	[80]
p‐Nitrotetrazolium blue	General detection of ROS by reduction of compound	Precipitation reaction (microscopy)	[188]
Oxyburst	General detection of ROS by oxidation of fluorophore	IF	[82, 189]
Phagosome crosstalk and signaling assays			
Anti‐EEA1	Early endosome marker (early maturation marker)	IF; EM	[126]
Anti‐LAMP1	Lysosome marker (late maturation marker)	IF; EM; flow cytometry	[51, 190]
Anti‐LAMP2	Lysosome marker (late maturation marker)	IF; EM	[191, 192]
Fixable fluorescent 10 kDa dextran	Prelabeling of endosomes/ lysosomes	Fluorescence‐based imaging	[193]
Fluorescent wheat germ agglutinin	Prelabeling of endosomes/ lysosomes	Flow cytometry	[41]
BSA‐gold conjugates	Prelabeling of endosomes/ lysosomes	EM	[194]
Tagged genetically encoded constructs	Fixed and live cells	Fluorescence‐based imaging	[187]
Anti‐ERGIC‐53/p58	ERGIC protein	IF	[126, 146]
Anti‐TGN46	TGN protein	IF	[146]
Anti‐TGN38	TGN protein	IF	[126]
Anticalreticulin	ER protein	IF	[146]
Antiprotein disulfide isomerase	ER enzyme	IF	[146]
mTOR	mTOR localization	IF	[114, 132]
Anti‐S6K	mTORC1 activation	Immunoblot	[23, 121]
TFEB/TF3	Translocation to nucleus	Immunoblot	[113]

The study of protein recruitment to individual phagosomes, along with their proteolytic activity, can also be achieved on isolated phagosomes by immunolabeling postnuclear homogenates of phagocytic cells after bead capture, followed by flow cytometry and gating on the bead population [67, 68, 69]. This approach allows the simultaneous detection of a wide array of proteins in a quantitative way. However, the number of parameters analyzed, albeit higher than in IF microscopy, is still limited by the fluorophore spectral overlap. In this regard, the recent development of mass cytometry has significantly increased the number of proteins that can be simultaneously quantified on a single‐cell level by using probes coupled to heavy‐metal isotopes instead of fluorophores, with little signal overlap between parameters [70].

#### Genetically encoded tools

While a carefully designed immunolabeling experiment can provide some temporal information on biological processes, live‐cell imaging provides the best strategy to improve resolution. The most suitable approach to do this is the expression of fluorescently tagged genetically encoded tools. Through this approach, researchers can follow the fate of molecules during dynamic processes such as phagocytosis. An added advantage of using these methods is that in addition to proteins, several lipid species can be monitored by expression of lipid‐sensing probes, which are designed from protein domains that bind a single lipid species with exquisite specificity and high sensitivity [71]. Several factors must be taken into consideration, most notably the fact that exogenous expression of proteins in most cases results in increased total expression of the protein of interest, which can result in diverse artifacts. Also, when expressing sensing probes, it is possible that they can outcompete binding of endogenous effectors and interfere with lipid metabolism and signaling. Thus, it is important to express these tools at levels as moderate as possible (i.e., close to those of endogenous proteins, as long as they are still detectable). This may be achieved by regulated viral vectors and/or conditional gene expression, such as the use of tetracycline‐dependent transcriptional switches and more recently developed optogenetic tools [72, 73]. Another limitation is that transfection/transduction of primary cells is significantly more challenging than that of transformed cell lines. However, several groups have optimized protocols (mainly using viral transduction) to study primary phagocytes [74, 75]. Another consideration is that while traditional live‐cell imaging techniques (i.e., confocal microscopy) provide suitable spatial and temporal resolution, techniques such as EM and super‐resolution microscopy (in most cases exclusively applicable to fixed cells) are superior to spatial resolution [66]. Alternative approaches for live‐cell imaging, albeit significantly more challenging, include endogenous tagging and the use of primary cells from transgenic mice [76].

#### ROS detection

During phagocytosis of microorganisms, phagocytes increase their oxygen consumption. In this process, the NADPH oxidase NOX2, transports electrons from cytosolic Nicotinamide adenine dinucleotide phosphate (NADPH) to the lumen of phagosomes where molecular oxygen is reduced to O_2_^–^, which is further converted into other highly microbicidal ROS [77, 78, 79]. Additionally, during inflammation, some phagocytes are capable of generating ROS extracellularly through activation of NADPH oxidase located at the plasma membrane [80]. ROS production plays additional roles in regulating phagosomal pH to preserve antigens for cross‐presentation in dendritic cells (DCs) and may also impact phagosome membrane integrity [34, 81, 82, 83], although pH‐independent proteolytic functions of NADPH oxidase have also been demonstrated [84]. Diverse methods have been developed to measure ROS production inside and outside of cells. In general, these techniques—which are typically specific to a particular oxygen species—are primarily based on ROS‐excitable dyes (e.g., luminol and isoluminol), substrate reduction (e.g., cytochrome c and *p*‐nitroblue tetrazolium), or fluorophore oxidation (*p*‐hydroxyphenylate, scopoletin, dichlorofluorescein, and dihydrorhodamine). These methods are detailed in various protocol guides adapted to the study of particular phagocytes [67, 80, 85].

#### pH measurement

Soon after the first description of phagocytosis, efforts were made to measure changes in phagosomal pH. Such methods that qualitatively determine pH changes have been commonly used in the field for the past ~ 60 years. The most common cellular and subcellular pH assessments rely on the use of indicator fluorophores such as the commercially available LysoTracker, pHrodo, and cresyl violet. Technically, phagocytic targets can be prelabeled covalently with one of such dyes (e.g., pHrodo) and then fed to phagocytes, in which changes in fluorescence intensity can be tracked over time. Alternatively, acidic phagosomes can be labeled with targeted fluorophores, typically weak bases that partition into acidic compartments and accumulate therein. While these techniques are useful and informative for rapid (and relatively easy) determination, they are purely qualitative. Proper quantitative measurements of phagosomal pH require more specialized methods. Dual‐excitation ratiometric imaging enables accurate and sensitive quantitative determinations at a subcellular level [86, 87]. This technique relies on the use of a fluorophore that has a highly pH‐sensitive excitation/emission wavelength peak and a second excitation/emission wavelength that is pH‐independent. Even though changes in fluorescence intensity of the pH‐sensitive wavelength reflect pH changes, they can also derive from photobleaching, dye leakage, or changes in focal plane. Hence, the second (pH‐insensitive) wavelength is used to normalize for these potential artifacts. Thus, the ratio of fluorescence intensities at different wavelengths is used to monitor exclusively changes in pH. A fundamental aspect of this method is that such ratiometric values can be converted into pH values through the use of a calibration curve. This curve is developed *in situ* with the use of pH calibration solutions containing ionophores in order to adjust the pH of intracellular compartments or cells and measure the ratios of the fluorophore of interest at specific pH values. For phagocytosis assays, targets can be prelabeled with fluorophores such as fluorescein and Oregon green, and pH‐insensitive fluorophores should also be included for ratiometric purposes [67, 88].

#### Proteolytic and bactericidal activity

Along their maturation, phagosomes acquire degradative capacity, which in most phagocytes is supported by the increased acidity in their luminal environment [7]. Particularly in DCs, proteolytic activity is specifically regulated and geared toward the presentation of the resulting antigenic peptides to T cells [89]. Protein cargo degradation in phagosomes is generally assessed by the use of ovalbumin (OVA) or BSA‐coated phagocytic targets, or cross‐linked to red blood cells (RBC). OVA degradation on phagosomes may be monitored over time after phagocytosis by flow cytometry, using anti‐OVA antibodies or fluorophore‐conjugated OVA. The evaluation of protein degradation on isolated phagosomes is performed by comparing the initial fluorescent peak corresponding to undegraded OVA (high mean fluorescence intensity, MFI) to the decrease in MFI observed over time as OVA is degraded during the phagosome maturation process [67, 68]. Another frequently used reagent to evaluate phagosome proteolytic capacity is DQ‐BSA, a self‐quenched fluorophore—DQ™ Green or DQ Q™ Red—conjugated to BSA. This assay is based on the cleavage of DQ‐BSA on the surface of phagocytic particles, by proteases present in the maturing phagosome, which leads to the generation of fluorescent products that can be analyzed by fluorescence microscopy [90, 91, 92]. More specific proteolytic activity measurements can be performed on isolated phagosomes at different maturation stages, by incubating phagosome extracts with fluorogenic cathepsin substrates and monitoring substrate degradation by fluorometric analyses [51, 93].

Phagosomal bactericidal activity can be monitored over time after bacteria uptake, by sequentially allowing phagocytosis to occur, killing extracellular bacteria with gentamicin, lysing phagocytes, and plating cell lysates to count bacterial colonies as a measure of bacteria viability [94]. Replicating bacteria within phagocytes may also be detected by flow cytometry by colabeling bacteria with proliferation dyes (such as eFluor Q™) and regular fluorochromes. When bacteria replicates, the proliferation dye becomes increasingly diluted among daughter cells and is eventually undetected, while the regular dye remains constant. MFI ratio between both dyes are calculated over time to assess bacteria replication and may be compared between different phagocytes and/or cell treatments [95].

## Chapter 2. Additional methods to assess phagosome maturation‐associated pathways

### Phagosome tubulation and crosstalk

#### At a glance

A striking transformation that some phagosomes undergo along their maturation pathway is the extension of phagosomal membrane tubules (phagotubules), which play different important roles according to the nature of the phagocyte. In DCs, phagotubules favor interphagosomal crosstalk and major histocompatibility complex (MHC)–class II antigen presentation [96]. Phagotubules may also favor the stimulation of pattern recognition receptors (PRR) in the cytosol by increasing the available surface for potential phagosomal leakage [97] (Fig. 2). In macrophages, the formation of distinct phagotubules serves different roles: Early on, it allows recycling of plasmalemmal components; at late stages, it promotes phagolysosomal formation and requires the association of phagosomes with microtubule‐associated motor proteins [27]; and finally, as described in the third chapter of this guide, phagotubules are associated with phagosome resorption and resolution [23, 24]. While the nature and role of phagotubules at different stages of the life cycle of phagosomes and also between phagocytes likely differ, the current lack of specific methodologies to study the diversity of phagotubules has limited their examination to general imaging techniques. Because of this, new methodological approaches are required for the unequivocal assessment of phagotubule identity and function, which will undoubtedly advance the understanding of these dynamic events.

### Imaging and fluorescence‐based methods

Given the dynamic nature of these events, the preferred method for the detection of phagosomal tubulation is live‐cell imaging [24, 98, 99]. As a less efficient but in some cases more accessible alternative, phagotubules may also be visualized on fixed cells by IF or immunoelectron microscopy [27]. However, given their transient nature, it is recommended to use conditions that ensure phagotubule stability after fixation. These conditions are often analogous to the ones required to preserve the integrity of microtubules [100, 101]. In our hands, the use of a periodate–lysine–paraformaldehyde fixative [102] followed by permeabilization with 0.1% saponin proved to be successful for the detection of OVA‐containing phagotubules by fluorescence microscopy (manuscript in revision).

Phagosomal tubulation may also potentially favor the interaction between phagosomes and other organelles. These interactions can be detected by IF or immunoelectron microscopy, as discussed above. Flow cytometry also proved successful for the detection of phagosomal crosstalk among phagosomes carrying a Toll‐like receptor (TLR) signature [96]. The same principle may be applied to the study of phagosomal recruitment of fluorescently tagged proteins present in different organelles. Of note, caution should be exerted when studying interorganelle membrane contact sites (MCS). Nonfunctional close proximity between organelles is common in the cytosol; thus, contaminants are often found when performing biochemical methods, and imaging techniques can result in artifacts, both of which can be misinterpreted as functional crosstalk. Thus, the use of complementary approaches is highly recommended to study MCS, in addition to the design of critical functional assays (e.g., identification of membrane tethers and their manipulation) [19, 24, 103, 104]

### Phagosomal PRR signaling and MHC‐II presentation

#### At a glance

Phagosomes are autonomous signaling organelles equipped with the necessary machinery for protein degradation, peptide loading, and subsequent antigen presentation on MHC‐II molecules [68, 105, 106, 107, 108]. At the same time, signaling from PRR stimulated on phagosomes favors phagosome maturation and leads to the production of proinflammatory cytokines that shape the outcome of the immune response [5, 109]. Furthermore, receptor‐mediated phagocytosis or PRR signaling from phagosomes may also lead to the activation of the transcription factors TFEB and TFE3—master regulators of lysosomal biogenesis and function, and autophagy [110, 111]—via mechanistic target of rapamycin (mTOR)‐dependent and mTOR‐independent mechanisms, enhancing phagosome degradative capacity and upregulating the transcription of proinflammatory and antimicrobial gene signatures (Fig. 2) [112, 113, 114] and thoroughly reviewed in Ref. [16].

The process of antigen MHC‐II presentation has been comprehensively reviewed, and we direct readers to some examples of this excellent and extensive literature [115, 116, 117, 118, 119, 120]. Given that we will refer to tools for the study of antigen presentation and recognition by T cells, we would like to point out that the standardized nomenclature for rat and mouse MHC can be found at the Jackson Laboratory homepage (http://www.informatics.jax.org/mgihome/nomen/). In particular, mouse MHC molecules are referred to as histocompatibility 2 (H2), and in the case of mouse MHC‐II molecules H2‐I‐A or H2‐I‐E, the designation is frequently shortened to I‐A or I‐E followed by a superscript denoting the haplotype. In the case of human MHC, these molecules are designated as human leukocyte antigens (HLA; see below and Table 2). The standardized nomenclature is periodically revised by the World Health Organization and can be accessed via the international ImMunoGeneTips project/HLA database (https://www.ebi.ac.uk/ipd/imgt/hla/).

**Table 2:** Selected reagents used in phagosome maturation‐associated pathways and resolution.

Reagent	Description	Applications	References
Antigen class II presentation			
Eα_52‐68_:I‐A^b^ YAe antibody	Antibody to peptide:MHC‐II complex	IF; flow cytometry	[124, 195]
1H3.1 mouse	TCR specific to Ealpha_52‐68_:I‐A^b^	Immunoassays	[124]
OT‐II mouse	TCR specific to OVA_323‐339_:IA^b^	Immunoassays	[123]
CN.B1 mouse and T‐cell clone	TCR specific to STm flagellin FliC_427‐441_:I‐A^b^	Immunoassays	[129]
CBir1Tg mouse and T‐cell clone	TCR specific to CBir flagellin_456–475_: I‐A^b^	Immunoassays	[130]
Antigen cross‐presentation			
OVA_257‐264_:25D1.16 antibody	Antibody to peptide:MHC‐I complex	IF; flow cytometry	[126]
Influenza_365‐380_:H‐2D^b^ antibody	Antibody to peptide:MHC‐I complex	IF; flow cytometry	[154, 155]
HSV_498‐505_:H‐2K^b^ antibody	Antibody to peptide:MHC‐I complex	IF; flow cytometry	[156]
OT‐I mouse	TCR specific to OVA_257‐264_:H‐2K^b^	Immunoassays	[150]
B3Z hybridoma	TCR specific to OVA_257‐264_:H‐2K^b^	Immunoassays	[153]
OGDH hybridoma	TCR specific to OGDH:H‐2K^b^	Immunoassays	[157]
HSV hybridoma	TCR specific to HSV_498‐505_:H‐2K^b^	Immunoassays	[156]
gp100 human T‐cell clone	TCR specific to peptide:HLA‐A2	Immunoassays	[151]
MART1 human T‐cell clone	TCR specific to peptide:HLA‐A2	Immunoassays	[151]
Phagosome integrity			
FITC/TRITC dextran	Leakage to cytosol	IF	[164]
N‐glycosylated Renilla luciferase	Enzymatic reaction in the cytosol	Luminescence	[158, 159]
FRET probe CCF4 and β‐lactamase	Enzymatic reaction in the cytosol	Fluorescence microscopy/flow cytometry	[83, 146, 170]
Anti‐Galectin‐3 antibodies	Membrane damage detection	IF	[167, 169]
Anti‐Galectin 8	Membrane damage detection	IF; immunoblot	[167, 169]
Antibodies against ESCRT components	Membrane damage repair	IF	[167]
Phagosome resolution			
5‐ (and 6‐) carboxytetramethylrhodamine succinimidyl ester	Phagolysosomal fission/ fragmentation	Lysosome reformation	[24]

### Biochemical assays

Phagocytosis and phagosome maturation‐induced PRR signaling can be readily assessed by immunoblotting of whole‐cell lysates at different time points after engulfment. Given that cell surface PRRs are initially triggered, a time course over phagocytosis together with a control with soluble PRR ligand is required to differentiate between plasmalemmal and phagosome‐intrinsic PRR signaling. The detection of a second wave of phosphorylation of kinases present on the TLR pathway (such as p38) can be easily detected by immunoblotting [51]. This strategy can be applied to different PRR (or other phagosomal receptors of interest) signaling pathways.

With regard to the study of phagosome–lysosome crosstalk and signaling along the maturation process, lysosomal mTOR complex 1 (mTORC1) activation can be measured by immunoblotting for changes in mTORC1 substrates such as the phosphorylation of the ribosomal S6 kinase 1 [121]. Activation of TFEB and TFE3 can also be monitored by assessing their phosphorylation status and nuclear translocation by cellular fractionation and immunoblotting [113].

### Fluorescence‐based methods

MHC‐II presentation from phagocytosed cargo can be assessed by IF microscopy and flow cytometry. Widely used procedures involve protein‐coated polystyrene beads as phagocytic cargo and antibodies that recognize peptide:MHC‐II complexes such as the pair Eα_52‐68_:I‐A^b^/ YAe antibody and others more recently developed [122]. Indirect readouts for MHC‐II presentation include monitoring the cell surface expression of activation markers on T‐cell clones specific to certain peptide:MHC‐II complexes, such as murine OT‐II (reactive to OVA_323‐339_:I‐A^b^) [123] or 1H3.1 (reactive to Eα_52‐68_:I‐A^b^) [124] T cells, or tetanus toxoid‐specific human T cells [125]. The use of OVA or viral protein‐expressing bacteria such as *Escherichia coli*, *Listeria monocytogenes,* or *Salmonella* typhimurium (STm) [126, 127, 128] as phagocytic cargo, followed by the coculture with antigen‐specific T‐cell clones, is also possible. However, the detection of MHC‐II presentation of bacterial antigens would be desirable and more informative in terms of the evaluation of bacterial infections and host–pathogen interactions. In this regard, there are some available T‐cell clones such as CN.B1 (reactive to STm flagellin FliC_427‐441_) [129] and T‐cell receptor (TCR) transgenic mice such as CBir1Tg (specific to commensal intestinal bacteria flagellin) [130] and CN.B1 (specific to STm flagellin) [129]. Moreover, novel multiparametric flow cytometric‐based approaches also allow the analysis of the expression on MHC‐II molecules of different pathogen‐specific antigens at the same time, by the use of various T‐cell hybridomas expressing unique fluorescent reporters [131] (Table 2).

The study of the crosstalk between phagosomes and lysosomes can be assessed by IF microscopy to monitor mTORC1 localization to lysosomes or phagosomes and TFEB/TFE translocation to the nucleus [113, 114, 132].

### Immunoassays

The production of cytokines after phagocyte stimulation by the phagocytic cargo (e.g., proinflammatory cytokines such as IL‐6, IL‐12, and Tumor necrosis factor alpha) [133] or by phagosomal damage [Interleukin‐1 beta (IL‐1β); see below] or after subsequent T‐cell activation (such as IL‐2) can be assessed by ELISA on fresh or frozen culture supernatant from stimulated cells. This method can be also applied to the activation of T‐cell hybridomas, such as OT4H.1D5 and OT4H.2D5, specific to OVA_265–280_:I‐A^b^ [134]; 3A9, specific to hen egg lysozyme_48‐62_:I‐A^k^ [135]; or various available T‐cell hybridomas, specific to influenza hemagglutinin protein [136]. Most of these hybridomas contain the LacZ gene downstream the IL‐2 promoter. Therefore, upon T‐cell activation, β galactosidase can also be measured colorimetrically [137] in an easy and cost‐effective—though less sensitive compared with other methods—way. Cytokine detection may also be accomplished by flow cytometry, which provides information about differences in the cell population. In the traditional setup, intracellular cytokines are detected after fixation and permeabilization. A variant of this method relies on their detection in live cells by using a matrix that retains cytokines on the cell surface. The advantage of this improved flow cytometric approach is that cells remain viable for further studies [138, 139]. However, ELISA provides greater sensitivity, the possibility to quantify the amount of the cytokines produced by the population and the option of assessing the samples at the most convenient time and on repeated occasions. Alternatively, the presence of cytokines or chemokines in culture supernatants can be detected by the use of commercially available cytokine dot‐blot arrays, which confer the advantage of detecting multiple cytokines simultaneously albeit in a semiquantitative manner [113].

### Antigen cross‐presentation

#### At a glance

Antigen cross‐presentation consists in the presentation of internalized exogenous antigens on MHC‐I molecules and is particularly relevant for the activation of cytotoxic Cluster of differentiation 8 protein (CD8)^+^ T cells in the development of an antiviral or antitumoral immune response [140, 141, 142]. In the case of phagocytosis, the process starts by the engulfment of targets and takes place more efficiently in a subset of DCs specialized in this mechanism [143, 144]. Antigen cross‐presentation is then mainly accomplished through the ‘cytosolic’ pathway. In this pathway, phagosomal antigens that gain access to the cytosol—by mechanisms currently not completely elucidated [145]—are degraded by the proteasome, and the resulting peptides are loaded onto MHC‐I molecules within the ER.

Increasing evidence supports the notion that phagosomes become competent for antigen cross‐presentation after delivery of MHC‐I molecules and other ER‐resident proteins from the ER, ER–Golgi intermediate compartment (ERGIC), or the endosomal recycling compartment (ERC) [105, 126, 146]. More recently, it has been proposed that phagosomes contain active proteasomes capable of generating intraphagosomal antigenic peptides and rendering phagosomes self‐sufficient antigen cross‐presentation organelles [147].

### Fluorescence‐based methods

Regardless of the relative contribution of the ER or the ERC to the phagosome maturation process, which remains controversial [104, 148], these events are mostly being analyzed by IF and live‐cell imaging. Antibodies to resident ER, ERGIC, or ERC compartments suitable for IF, as well as fluorescently tagged organelle markers, are readily available (Table 1).

Like MHC‐II presentation, antigen cross‐presentation from phagosomes can be assessed by IF microscopy and flow cytometry using protein‐coated polystyrene beads as cargo and antibodies that recognize peptide:MHC‐I complexes, such as anti‐OVA_257‐264_:H‐2K^b^ [149].

To evaluate T‐cell activation after antigen cross‐presentation, cell surface expression of activation markers is assessed on T‐cell clones specific to certain peptide:MHC‐I complexes, such as murine OT‐I, reactive to OVA_257‐264_:H‐2K^b^ [150]; or gp91, or melanoma‐specific human T cells [151, 152].

### Immunoassays

The production of IL‐2 from activated T cells can be measured by ELISA as described for MHC‐II presentation assays. This also applies to the use of T‐cell hybridomas B3Z,specific to OVA_257‐264_:H‐2K^b^ [153]; hybridoma that recognizes influenza nucleoprotein_365‐380_:H‐2D^b^ [154, 155]; HSV‐2.3.2E2 that recognizes herpes simplex virus glycoprotein B_498‐505_:H‐2K [156]; or 2CZ that recognizes oxoglutarate dehydrogenase peptide:H‐2K [157]. Despite being simple and rapid to test, the downside of using hybridomas and some Tcell clones resides mainly in the maintenance of cumbersome culture techniques and complex growing conditions.

### Luminescence methods

A novel luciferase‐based probe was designed to assess antigen translocation to the cytosol [158, 159], in most cases required for proteasomal degradation and subsequent peptide loading in the cross‐presentation process. The probe consists in an enzymatically inactive N‐glycosylated variant of Renilla luciferase fused to the Fragment crystallizable (Fc) region of human IgG1. This variant becomes enzymatically active when deglycosylated by the cytosolic enzyme N‐glycanase‐1, generating cytosolic luminescence, which can be easily quantified. This strategy has the potential to be adapted to fusion proteins of interest and can be targeted to other phagocytic receptors as well.

Antigen translocation or exit to the cytosol can also be predicted by assessing phagosome integrity as will be discussed below.

### Phagosome integrity

#### At a glance

Some phagocytic cargoes, such as cholesterol crystals, alum used in vaccine adjuvants, and bacterial pathogens may compromise phagosome integrity by causing membrane destabilization or active membrane damage [160]. Alternatively, lipid peroxidation mediated by the NADPH oxidaseNOX2, may also lead to membrane integrity disruption [83]. When phagosome integrity is compromised, phagosomal content can gain access to the cytosol and trigger additional cellular processes and signaling cascades, such as inflammasome activation, autophagy induction, and/or antigen cross‐presentation (Fig. 2). Moreover, compromising the integrity of the phagosomal membrane and the escape of its contents can ultimately result in cell death. Thus, the assessment of phagosome integrity is relevant to the study of the nature of the engulfed particle, the threat it potentially poses to the phagocyte, the cellular mechanisms activated by phagosome damage, and the type of immune response consequently triggered.

### Biochemical methods

Phagosomal acquisition of proteins associated with membrane damage and/or repair (see below) may be assessed by immunoblotting [161] on isolated phagosomes. Controls for purity of phagosome preparations are required.

The production of active IL‐1β—released if inflammasome activation is triggered after phagosome damage—compared to the pro‐IL‐1β form—triggered by inflammatory cargo in an intact phagosome—can be assessed by immunoblotting as an alternative indirect measure of phagosome integrity that also provides information about downstream inflammasome activity in the cytosol [162].

### Fluorescence‐based methods

Phagosome integrity can be measured by fluorescence microscopy approaches in live or fixed cells. One of these approaches is based on the dextran‐release assay, which relies on the quantification of fluorescently labeled‐dextran in the cytosol after phagolysosome formation and damage [163]. Phagocytic cells can also be preloaded with two discernible dextran–fluorophore conjugates as endolysosomal cargoes and then pulsed with a particulate phagosomal prey [164]. In this case, when lysosomes fuse with phagosomes in the process of phagosome maturation, dextran release to the cytosol (in the case of phagosomal damage) is quantified by ratiometric imaging between the two fluorophores. Alternatively, fluorescent dextran can be adsorbed to or loaded into some phagosomal cargoes (such as RBC) [165, 166]. In this case, the assay can directly evaluate phagosomal leakage and becomes independent of lysosomal contribution. Another approach that is independent from phagosome maturation is the quantification of the recruitment of proteins that mark damaged membranes, such as galectins [167]. Galectins are cytosolic lectins that bind galactosides present on the luminal leaflet of organellar membranes and can therefore bind to phagosomal membranes when galactosides are exposed to the cytosol after membrane damage. Recruitment of galectins 3 and 8 to phagosomal membranes has been monitored by fluorescence microscopy [167, 168, 169]. Automated quantitative imaging of fluorescent puncta may be performed using high‐content analysis platforms [167]. Binding of galectins to phagosomal membranes can also lead to the recruitment of proteins involved in membrane repair, such as Endosomal sorting complexes required for transport (ESCRT) complexes, or in damaged organelle removal, such as autophagy receptors or adaptors. Recruitment of ESCRT proteins and autophagy proteins, such as Microtubule‐associated proteins 1A/1B light chain 3 and p62, can be assessed by fluorescence microscopy as an indirect measurement of phagosome damage [169].

Another possibility is based on a method used to measure escape to the cytosol of β‐lactamase‐expressing bacteria [170] and has also been applied to the study of antigen export to the cytosol in the antigen cross‐presentation field [146, 171]. The assay consists of preloading cells with the fluorescence resonance energy transfer (FRET) probe CCF4 prior to phagocytosis. When β‐lactamase is present in the cytosol, it cleaves the probe, resulting in a loss of FRET signal at 535 nm and an increased emission at 450 nm, which can be quantified by fluorescence microscopy or flow cytometry.

Phagosome integrity after bacterial infection can also be assessed by flow cytometry, based on the assay described to quantify cytosolic versus vacuolar STm by differential permeabilization [172]. Based on the differences in abundance of cholesterol between the plasma membrane and intracellular organelles, treatment with digitonin under standardized conditions of time and concentration exclusively permeabilizes the plasma membrane, while saponin permeabilizes both plasma membrane and intracellular organelles. This assay can be extended to any phagosomal cargo, provided that the cargo (or a chemically modified version of it) can be detected by antibodies. If the phagosome is intact, the cargo will not be detected by antibodies in the presence of digitonin, but it will be detected in the presence of saponin. Conversely, if the phagosome is damaged, the antibodies will detect the cargo in the presence of either digitonin or saponin. Various degrees of detection (according to the level of availability of the cargo to the antibodies) over time after phagocytosis can be quantified by flow cytometry and normalized to the total detection levels in the presence of saponin. Due to the requirement to maintain the conditions that prevent digitonin to permeabilize intracellular membranes, controls for the detection of intraorganellar and cytosolic epitopes such as ER proteins are essential to validate the assay.

## Chapter 3. Methods to assess phagosome resolution

### Phagosome resolution

#### At a glance

The biochemical composition of physiological phagocytic targets is usually complex. While phagolysosomes are equipped with a wide array of hydrolases to metabolize most macromolecules, the catabolites of this breakdown must be processed for the cell to resorb the compartment, recycle its components, and return to homeostasis in order to resume the immune response. Additionally, it is through processing of degradation products that antigen is presented on the surface of specialized phagocytes to lymphoid cells (as detailed in Chapter 2). Despite its obvious physiological significance, the resolution of phagosomes has rarely been studied and a comprehensive understanding of the molecular mechanisms that drive it is still lacking. Because interest in resolution emerged recently, the methods to study this stage are under development. Some of the techniques that have been used are adapted from studies of lysosomes and autophagy. Here, we discuss methodologies that have been used in a handful of studies on phagosome resolution (excluding antigen presentation, which is discussed above).

### Biochemical methods

#### Detection of mTORC1

While phagosome resolution is by far the least understood stage of phagocytosis, some of its general aspects have been elucidated. Complete resorption of the phagosomal compartment is dependent on mTORC1. Indeed, the fission events that are necessary for membrane recycling and lysosome reformation are impaired upon mTORC1 inhibition [23]. Catabolite export from degraded cargo can promote mTORC1 activation, potentially promoting fission events. Thus, mTORC1 activation can be assessed as a proxy for the initiation of phagosome resolution. The caveat to this assessment is that mTORC1 should remain inactive during phagocytosis in order to detect significant changes in mTORC1 activation. To achieve this, cells can be amino acid‐deprived before phagocytes are challenged with degradable phagocytic targets and for the duration of the experiment [23]. mTORC1 activation will occur when catabolites (such as specific free amino acids, e.g., leucine) resulting from robust target degradation are exported from the phagolysosome. These events can be measured by immunoblotting for changes in mTORC1 substrates as discussed in Chapter 2. However, while this method can be used as a readout of phagosome resolution, it does not represent a common physiological state of phagocytes. This emphasizes the need for the development of new methodologies that more closely represent phagocyte host environment.

### Imaging and fluorescence‐based methods

#### Phagotubule formation

During the very late stage of phagocytosis, the original phagosomal compartment undergoes a series of fission events mediated at least in part by robust membrane tubulation. Methods to study phagosomal tubulation have been described above (phagosome tubulation and crosstalk section). More recently, we have used lattice light‐sheet microscopy (LLSM) to visualize dynamic phagosome tubulation [24]. Because frequent and continuous frame acquisition is critical to study these structures, LLSM minimizes photobleaching while enabling rapid acquisition of multiple focal planes. It is worth noting that this type of microscopy is highly specialized and not readily available yet to most researchers.

#### Cytosolic dispersion of phagosome‐derived vesicles

One of the hypothesized consequences of phagosome resorption during the resolution stage is the reformation of terminal/ storage lysosomes, as in the case of autophagic lysosome reformation [173, 174]. Thus, the above‐mentioned fission events result in the dispersion of smaller compartments (‘recovered’ organelles) throughout the cytoplasm. This phenomenology has served as a proxy to assess resolution, as researchers can challenge phagocytes with prelabeled (degradable) phagocytic targets (e.g., RBC and apoptotic cells), with pH‐insensitive dyes. After internalization, phagocytes are incubated for long time periods (at least 3–8 h) allowing phagocytosis to progress through resolution. Completion of the process can be measured through fluorescence microscopy by assessing the level of dispersion and size of vesicles of phagosomal origin [23, 24, 175]. Recent unpublished studies suggest that lysosome and/or endolysosome regeneration indeed occurs at the phagosome resolution stage, by the use of assays to detect lysosome proteins, pH, and proteolytic activity on phagosome‐derived vesicles. A more detailed description of these methods is accessible at: https://doi.org/10.1101/2020.05.14.094722.

## Conclusions

The study of phagosome maturation and resolution interfaces with different fields within the biological sciences, including cell biology, immunology, and microbiology, and has recently returned to the spotlight with the increased interest in defining MCS and organelle crosstalk. The study of phagosome dynamics also bridges biological and physicochemical areas of expertise by the continuous development of new tools and technologies for visualization and quantification of phagosome‐interrelated phenomena with increased precision, resolution, and sensitivity.

In this guide, we summarized some of the biochemical, imaging, fluorescence, luminescence, and immune‐based methods currently available and widely used in the literature for the study of phagosomal dynamics, with a main focus on two subset of phagocytes: macrophages and DCs, an arbitrary decision motivated by our areas of expertise. We also aimed at describing methodologies to integrate the process of phagosome maturation with other downstream cellular signaling pathways, such as inflammasome activation, mTORC1 signaling, and autophagy induction. Additionally, we offered our point of view on the advantages and limitations of diverse methods in an effort to help researchers in their experimental design. Moreover, we attempted to point out some of the areas of study where new or improved methodology would be desired to advance current knowledge.

Ultimately, by providing information about methods applied in different fields to the study of phagosome maturation and resolution, we intended to highlight the crucial role played by phagosomes at the crossroads of fundamental cellular processes.

## Conflict of interest

The authors declare no conflicts of interest.

## Author contributions

RL‐K and ARM wrote and revised the manuscript and designed the figures.

## References

[1] Metchnikoff E (1968) Lectures on the Comparative Pathology of Inflammation, Delivered at the Pasteur Institute in 1891. Dover Publications, New York, NY.

[2] Underhill DM , Gordon S , Imhof BA , Nunez G & Bousso P (2016) Elie Metchnikoff (1845–1916): celebrating 100 years of cellular immunology and beyond. Nat Rev Immunol 16, 651–656. 27477126 10.1038/nri.2016.89

[3] Underhill DM & Goodridge HS (2012) Information processing during phagocytosis. Nat Rev Immunol 12, 492–502. 22699831 10.1038/nri3244 PMC5570470

[4] Blander JM & Sander LE (2012) Beyond pattern recognition: five immune checkpoints for scaling the microbial threat. Nat Rev Immunol 12, 215–225. 22362354 10.1038/nri3167

[5] Kagan JC & Iwasaki A (2012) Phagosome as the organelle linking innate and adaptive immunity. Traffic 13, 1053–1061. 22577865 10.1111/j.1600-0854.2012.01377.x PMC3658133

[6] Levin R , Grinstein S & Canton J (2016) The life cycle of phagosomes: formation, maturation, and resolution. Immunol Rev 273, 156–179. 27558334 10.1111/imr.12439

[7] Flannagan RS , Jaumouille V & Grinstein S (2012) The cell biology of phagocytosis. Annu Rev Pathol 7, 61–98. 21910624 10.1146/annurev-pathol-011811-132445

[8] Jaumouille V & Grinstein S (2016) Molecular mechanisms of phagosome formation. Microbiol Spectr 4(3). 10.1128/microbiolspec.MCHD-0013-2015 27337463

[9] Niedergang F & Grinstein S (2018) How to build a phagosome: new concepts for an old process. Curr Opin Cell Biol 50, 57–63. 29471269 10.1016/j.ceb.2018.01.009

[10] Straus W (1964) Cytochemical observations on the relationship between lysosomes and phagosomes in kidney and liver by combined staining for acid phosphatase and intravenously injected horseradish peroxidase. J Cell Biol 20, 497–507. 14128050 10.1083/jcb.20.3.497 PMC2106412

[11] Steinman RM & Cohn ZA (1972) The interaction of particulate horseradish peroxidase (HRP)‐anti HRP immune complexes with mouse peritoneal macrophages *in vitro* . J Cell Biol 55, 616–634. 4656704 10.1083/jcb.55.3.616 PMC2108816

[12] Vieira OV , Botelho RJ & Grinstein S (2002) Phagosome maturation: aging gracefully. Biochem J 366, 689–704. 12061891 10.1042/BJ20020691 PMC1222826

[13] Kinchen JM & Ravichandran KS (2008) Phagocytic signaling: you can touch, but you can't eat. Curr Biol 18, R521–R524. 18579095 10.1016/j.cub.2008.04.058 PMC2851548

[14] Fairn GD & Grinstein S (2012) How nascent phagosomes mature to become phagolysosomes. Trends Immunol 33, 397–405. 22560866 10.1016/j.it.2012.03.003

[15] Pauwels AM , Trost M , Beyaert R & Hoffmann E (2017) Patterns, receptors, and signals: regulation of phagosome maturation. Trends Immunol 38, 407–422. 28416446 10.1016/j.it.2017.03.006 PMC5455985

[16] Hipolito VEB , Ospina‐Escobar E & Botelho RJ (2018) Lysosome remodelling and adaptation during phagocyte activation. Cell Microbiol 20, e12824. 10.1111/cmi.12824 29349904

[17] Mayorga LS , Bertini F & Stahl PD (1991) Fusion of newly formed phagosomes with endosomes in intact cells and in a cell‐free system. J Biol Chem 266, 6511–7. 2007600

[18] Desjardins M , Huber LA , Parton RG & Griffiths G (1994) Biogenesis of phagolysosomes proceeds through a sequential series of interactions with the endocytic apparatus. J Cell Biol 124, 677–688. 8120091 10.1083/jcb.124.5.677 PMC2119957

[19] Nunes‐Hasler P & Demaurex N (2017) The ER phagosome connection in the era of membrane contact sites. Biochim Biophys Acta Mol Cell Res 1864, 1513–1524. 28432021 10.1016/j.bbamcr.2017.04.007

[20] West AP , Brodsky IE , Rahner C , Woo DK , Erdjument‐Bromage H , Tempst P , Walsh MC , Choi Y , Shadel GS & Ghosh S (2011) TLR signalling augments macrophage bactericidal activity through mitochondrial ROS. Nature 472, 476–480. 21525932 10.1038/nature09973 PMC3460538

[21] Geng J , Sun X , Wang P , Zhang S , Wang X , Wu H , Hong L , Xie C , Li X , Zhao H *et al*. (2015) Kinases Mst1 and Mst2 positively regulate phagocytic induction of reactive oxygen species and bactericidal activity. Nat Immunol 16, 1142–1152. 26414765 10.1038/ni.3268 PMC4618176

[22] Griffiths G & Mayorga L (2007) Phagosome proteomes open the way to a better understanding of phagosome function. Genome Biol 8, 207. 17367543 10.1186/gb-2007-8-3-207 PMC1868947

[23] Krajcovic M , Krishna S , Akkari L , Joyce JA & Overholtzer M (2013) mTOR regulates phagosome and entotic vacuole fission. Mol Biol Cell 24, 3736–3745. 24088573 10.1091/mbc.E13-07-0408 PMC3842999

[24] Levin‐Konigsberg R , Montano‐Rendon F , Keren‐Kaplan T , Li R , Ego B , Mylvaganam S , DiCiccio JE , Trimble WS , Bassik MC , Bonifacino JS *et al*. (2019) Phagolysosome resolution requires contacts with the endoplasmic reticulum and phosphatidylinositol‐4‐phosphate signalling. Nat Cell Biol 21, 1234–1247. 31570833 10.1038/s41556-019-0394-2 PMC8340083

[25] Moretti J & Blander JM (2014) Insights into phagocytosis‐coupled activation of pattern recognition receptors and inflammasomes. Curr Opin Immunol 26, 100–110. 24556406 10.1016/j.coi.2013.11.003 PMC3932007

[26] Blocker A , Severin FF , Burkhardt JK , Bingham JB , Yu H , Olivo JC , Schroer TA , Hyman AA & Griffiths G (1997) Molecular requirements for bi‐directional movement of phagosomes along microtubules. J Cell Biol 137, 113–129. 9105041 10.1083/jcb.137.1.113 PMC2139871

[27] Harrison RE , Bucci C , Vieira OV , Schroer TA & Grinstein S (2003) Phagosomes fuse with late endosomes and/or lysosomes by extension of membrane protrusions along microtubules: role of Rab7 and RILP. Mol Cell Biol 23, 6494–6506. 12944476 10.1128/MCB.23.18.6494-6506.2003 PMC193691

[28] Al‐Haddad A , Shonn MA , Redlich B , Blocker A , Burkhardt JK , Hanry Y , Hammer JA 3rd , Weiss DG , Steffen W & Griffiths G (2001) Myosin Va bound to phagosomes binds to F‐actin and delays microtubule‐dependent motility. Mol Biol Cell 12, 2742–2755. 11553713 10.1091/mbc.12.9.2742 PMC59709

[29] Anes E , Kuhnel MP , Bos E , Moniz‐Pereira J , Habermann A & Griffiths G (2003) Selected lipids activate phagosome actin assembly and maturation resulting in killing of pathogenic mycobacteria. Nat Cell Biol 5, 793–802. 12942085 10.1038/ncb1036

[30] Vieira OV , Botelho RJ , Rameh L , Brachmann SM , Matsuo T , Davidson HW , Schreiber A , Backer JM , Cantley LC & Grinstein S (2001) Distinct roles of class I and class III phosphatidylinositol 3‐kinases in phagosome formation and maturation. J Cell Biol 155, 19–25. 11581283 10.1083/jcb.200107069 PMC2150784

[31] Levin R , Grinstein S & Schlam D (2015) Phosphoinositides in phagocytosis and macropinocytosis. Biochim Biophys Acta 1851, 805–823. 25238964 10.1016/j.bbalip.2014.09.005

[32] Savina A , Peres A , Cebrian I , Carmo N , Moita C , Hacohen N , Moita LF & Amigorena S (2009) The small GTPase Rac2 controls phagosomal alkalinization and antigen crosspresentation selectively in CD8(+) dendritic cells. Immunity 30, 544–555. 19328020 10.1016/j.immuni.2009.01.013

[33] Canton J , Khezri R , Glogauer M & Grinstein S (2014) Contrasting phagosome pH regulation and maturation in human M1 and M2 macrophages. Mol Biol Cell 25, 3330–3341. 25165138 10.1091/mbc.E14-05-0967 PMC4214780

[34] Kotsias F , Hoffmann E , Amigorena S & Savina A (2013) Reactive oxygen species production in the phagosome: impact on antigen presentation in dendritic cells. Antioxid Redox Signal 18, 714–729. 22827577 10.1089/ars.2012.4557

[35] Paardekooper LM , Dingjan I , Linders PTA , Staal AHJ , Cristescu SM , Verberk W & van den Bogaart G (2019) Human monocyte‐derived dendritic cells produce millimolar concentrations of ROS in phagosomes per second. Front Immunol 10, 1216. 31191556 10.3389/fimmu.2019.01216 PMC6548834

[36] Wetzel MG & Korn ED (1969) Phagocytosis of latex beads by *Acahamoeba castellanii* (Neff). 3. Isolation of the phagocytic vesicles and their membranes. J Cell Biol 43, 90–104. 4309954 10.1083/jcb.43.1.90 PMC2107834

[37] Ulsamer AG , Wright PL , Wetzel MG & Korn ED (1971) Plasma and phagosome membranes of *Acanthamoeba castellanii* . J Cell Biol. 51, 193–215. 4329520 10.1083/jcb.51.1.193 PMC2108235

[38] Desjardins M , Celis JE , van Meer G , Dieplinger H , Jahraus A , Griffiths G & Huber LA (1994) Molecular characterization of phagosomes. J Biol Chem 269, 32194–32200. 7798218

[39] Rogers LD & Foster LJ (2007) The dynamic phagosomal proteome and the contribution of the endoplasmic reticulum. Proc Natl Acad Sci USA 104, 18520–18525. 18006660 10.1073/pnas.0705801104 PMC2141809

[40] Campbell‐Valois FX , Trost M , Chemali M , Dill BD , Laplante A , Duclos S , Sadeghi S , Rondeau C , Morrow IC , Bell C *et al*. (2012) Quantitative proteomics reveals that only a subset of the endoplasmic reticulum contributes to the phagosome. Mol Cell Proteomics 11 (M111), 016378. 22427703 10.1074/mcp.M111.016378 PMC3394953

[41] Alloatti A , Kotsias F , Pauwels AM , Carpier JM , Jouve M , Timmerman E , Pace L , Vargas P , Maurin M , Gehrmann U *et al*. (2015) Toll‐like receptor 4 engagement on dendritic cells restrains phago‐lysosome fusion and promotes cross‐presentation of antigens. Immunity 43, 1087–1100. 26682983 10.1016/j.immuni.2015.11.006

[42] Pauwels AM , Hartlova A , Peltier J , Driege Y , Baudelet G , Brodin P , Trost M , Beyaert R & Hoffmann E (2019) Spatiotemporal changes of the phagosomal proteome in dendritic cells in response to LPS stimulation. Mol Cell Proteomics 18, 909–922. 30808727 10.1074/mcp.RA119.001316 PMC6495253

[43] Zeichner SL (1983) Isolation and characterization of macrophage phagosomes containing infectious and heat‐inactivated *Chlamydia psittaci*: two phagosomes with different intracellular behaviors. Infect Immun 40, 956–966. 6852926 10.1128/iai.40.3.956-966.1983 PMC348145

[44] Lutz DA , Chen XM & McLaughlin BJ (1993) Isolation of the phagocytic compartment from macrophages using a paramagnetic, particulate ligand. Anal Biochem 214, 205–211. 8250224 10.1006/abio.1993.1478

[45] Sturgill‐Koszycki S , Schlesinger PH , Chakraborty P , Haddix PL , Collins HL , Fok AK , Allen RD , Gluck SL , Heuser J & Russell DG (1994) Lack of acidification in *Mycobacterium phagosomes* produced by exclusion of the vesicular proton‐ATPase. Science 263, 678–681. 8303277 10.1126/science.8303277

[46] Luhrmann A & Haas A (2000) A method to purify bacteria‐containing phagosomes from infected macrophages. Methods Cell Sci 22, 329–341. 11549946 10.1023/a:1017963401560

[47] Lonnbro P , Nordenfelt P & Tapper H (2008) Isolation of bacteria‐containing phagosomes by magnetic selection. BMC Cell Biol 9, 35. 18588680 10.1186/1471-2121-9-35 PMC2453110

[48] Herweg JA , Hansmeier N , Otto A , Geffken AC , Subbarayal P , Prusty BK , Becher D , Hensel M , Schaible UE , Rudel T *et al*. (2015) Purification and proteomics of pathogen‐modified vacuoles and membranes. Front Cell Infect Microbiol 5, 48. 26082896 10.3389/fcimb.2015.00048 PMC4451638

[49] Ramachandra L , Noss E , Boom WH & Harding CV (2001) Processing of *Mycobacterium tuberculosis* antigen 85B involves intraphagosomal formation of peptide‐major histocompatibility complex II complexes and is inhibited by live bacilli that decrease phagosome maturation. J Exp Med 194, 1421–1432. 11714749 10.1084/jem.194.10.1421 PMC2193679

[50] Grotzke JE , Harriff MJ , Siler AC , Nolt D , Delepine J , Lewinsohn DA & Lewinsohn DM (2009) The *Mycobacterium tuberculosis* phagosome is a HLA‐I processing competent organelle. PLoS Pathog 5, e1000374. 19360129 10.1371/journal.ppat.1000374 PMC2661020

[51] Mantegazza AR , Guttentag SH , El‐Benna J , Sasai M , Iwasaki A , Shen H , Laufer TM & Marks MS (2012) Adaptor protein‐3 in dendritic cells facilitates phagosomal toll‐like receptor signaling and antigen presentation to CD4(+) T cells. Immunity 36, 782–794. 22560444 10.1016/j.immuni.2012.02.018 PMC3361531

[52] Steinhauser C , Dallenga T , Tchikov V , Schaible UE , Schutze S & Reiling N . (2014) Immunomagnetic isolation of pathogen‐containing phagosomes and apoptotic blebs from primary phagocytes. Curr Protoc Immunol 105, 14.36.1–14.36.26. 24700322 10.1002/0471142735.im1436s105

[53] Garin J , Diez R , Kieffer S , Dermine JF , Duclos S , Gagnon E , Sadoul R , Rondeau C & Desjardins M (2001) The phagosome proteome: insight into phagosome functions. J Cell Biol 152, 165–180. 11149929 10.1083/jcb.152.1.165 PMC2193653

[54] Dill BD , Gierlinski M , Hartlova A , Arandilla AG , Guo M , Clarke RG & Trost M (2015) Quantitative proteome analysis of temporally resolved phagosomes following uptake via key phagocytic receptors. Mol Cell Proteomics 14, 1334–1349. 25755298 10.1074/mcp.M114.044594 PMC4424403

[55] Stuart LM , Boulais J , Charriere GM , Hennessy EJ , Brunet S , Jutras I , Goyette G , Rondeau C , Letarte S , Huang H *et al*. (2007) A systems biology analysis of the Drosophila phagosome. Nature 445, 95–101. 17151602 10.1038/nature05380

[56] Buschow SI , Lasonder E , Szklarczyk R , Oud MM , de Vries IJ & Figdor CG (2012) Unraveling the human dendritic cell phagosome proteome by organellar enrichment ranking. J Proteomics 75, 1547–1562. 22146474 10.1016/j.jprot.2011.11.024

[57] Dieckmann R , Gopaldass N , Escalera C & Soldati T (2008) Monitoring time‐dependent maturation changes in purified phagosomes from *Dictyostelium discoideum* . Methods Mol Biol 445, 327–337. 18425460 10.1007/978-1-59745-157-4_21

[58] Pathak D , Mehendale N , Singh S , Mallik R & Kamat SS (2018) Lipidomics suggests a new role for ceramide synthase in phagocytosis. ACS Chem Biol 13, 2280–2287. 29963848 10.1021/acschembio.8b00438 PMC6102644

[59] Lee JJ , Lim J , Gao S , Lawson CP , Odell M , Raheem S , Woo J , Kang SH , Kang SS , Jeon BY *et al*. (2018) Glutamate mediated metabolic neutralization mitigates propionate toxicity in intracellular *Mycobacterium tuberculosis* . Sci Rep 8, 8506. 29855554 10.1038/s41598-018-26950-z PMC5981324

[60] Beron W , Colombo MI , Mayorga LS & Stahl PD (1995) *In vitro* reconstitution of phagosome‐endosome fusion: evidence for regulation by heterotrimeric GTPases. Arch Biochem Biophys 317, 337–342. 7893147 10.1006/abbi.1995.1172

[61] Jahraus A , Tjelle TE , Berg T , Habermann A , Storrie B , Ullrich O & Griffiths G (1998) *In vitro* fusion of phagosomes with different endocytic organelles from J774 macrophages. J Biol Chem 273, 30379–30390. 9804802 10.1074/jbc.273.46.30379

[62] Becken U , Jeschke A , Veltman K & Haas A (2010) Cell‐free fusion of bacteria‐containing phagosomes with endocytic compartments. Proc Natl Acad Sci USA 107, 20726–20731. 21071675 10.1073/pnas.1007295107 PMC2996438

[63] Jeschke A , Zehethofer N , Lindner B , Krupp J , Schwudke D , Haneburger I , Jovic M , Backer JM , Balla T , Hilbi H *et al*. (2015) Phosphatidylinositol 4‐phosphate and phosphatidylinositol 3‐phosphate regulate phagolysosome biogenesis. Proc Natl Acad Sci USA 112, 4636–4641. 25825728 10.1073/pnas.1423456112 PMC4403170

[64] Blocker A , Severin FF , Habermann A , Hyman AA , Griffiths G & Burkhardt JK (1996) Microtubule‐associated protein‐dependent binding of phagosomes to microtubules. J Biol Chem 271, 3803–3811. 8631997 10.1074/jbc.271.7.3803

[65] Jahraus A , Egeberg M , Hinner B , Habermann A , Sackman E , Pralle A , Faulstich H , Rybin V , Defacque H & Griffiths G (2001) ATP‐dependent membrane assembly of F‐actin facilitates membrane fusion. Mol Biol Cell 12, 155–170. 11160830 10.1091/mbc.12.1.155 PMC30575

[66] Baranov MV , Olea RA & van den Bogaart G (2019) Chasing uptake: super‐resolution microscopy in endocytosis and phagocytosis. Trends Cell Biol 29, 727–739. 31227311 10.1016/j.tcb.2019.05.006

[67] Savina A , Vargas P , Guermonprez P , Lennon AM & Amigorena S (2010) Measuring pH, ROS production, maturation, and degradation in dendritic cell phagosomes using cytofluorometry‐based assays. Methods Mol Biol 595, 383–402. 19941126 10.1007/978-1-60761-421-0_25

[68] Hoffmann E , Kotsias F , Visentin G , Bruhns P , Savina A & Amigorena S (2012) Autonomous phagosomal degradation and antigen presentation in dendritic cells. Proc Natl Acad Sci USA 109, 14556–14561. 22908282 10.1073/pnas.1203912109 PMC3437883

[69] Hoffmann E , Pauwels AM , Alloatti A , Kotsias F & Amigorena S (2016) Analysis of phagosomal antigen degradation by flow organellocytometry. Bio Protoc 6, e2014. 10.21769/bioprotoc.2014 PMC5321520 28239620

[70] Spitzer MH & Nolan GP (2016) Mass cytometry: single cells, many features. Cell 165, 780–791. 27153492 10.1016/j.cell.2016.04.019 PMC4860251

[71] Hammond GR & Balla T (2015) Polyphosphoinositide binding domains: key to inositol lipid biology. Biochim Biophys Acta 1851, 746–758. 25732852 10.1016/j.bbalip.2015.02.013 PMC4380703

[72] Toettcher JE , Voigt CA , Weiner OD & Lim WA (2011) The promise of optogenetics in cell biology: interrogating molecular circuits in space and time. Nat Methods 8, 35–38. 21191370 10.1038/nmeth.f.326 PMC3024327

[73] Yamada M , Suzuki Y , Nagasaki SC , Okuno H & Imayoshi I (2018) Light control of the Tet gene expression system in mammalian cells. Cell Rep 25, 487–500 e6. 30304687 10.1016/j.celrep.2018.09.026

[74] Manel N & Littman DR (2010) RNAi in human monocyte‐derived dendritic cells using shRNA vectors. Protoc Exch. 101038/protex2010208.

[75] Bobadilla S , Sunseri N & Landau NR (2013) Efficient transduction of myeloid cells by an HIV‐1‐derived lentiviral vector that packages the Vpx accessory protein. Gene Ther 20, 514–520. 22895508 10.1038/gt.2012.61 PMC4105013

[76] Serebrenik YV , Sansbury SE , Kumar SS , Henao‐Mejia J & Shalem O (2019) Efficient and flexible tagging of endogenous genes by homology‐independent intron targeting. Genome Res 29, 1322–1328. 31239279 10.1101/gr.246413.118 PMC6673721

[77] Segal AW (2008) The function of the NADPH oxidase of phagocytes and its relationship to other NOXs in plants, invertebrates, and mammals. Int J Biochem Cell Biol 40, 604–618. 18036868 10.1016/j.biocel.2007.10.003 PMC2636181

[78] Klebanoff SJ , Kettle AJ , Rosen H , Winterbourn CC & Nauseef WM (2013) Myeloperoxidase: a front‐line defender against phagocytosed microorganisms. J Leukoc Biol 93, 185–198. 23066164 10.1189/jlb.0712349 PMC3545676

[79] Nunes P , Demaurex N & Dinauer MC (2013) Regulation of the NADPH oxidase and associated ion fluxes during phagocytosis. Traffic 14, 1118–1131. 23980663 10.1111/tra.12115

[80] Dahlgren C , Karlsson A & Bylund J (2019) Intracellular neutrophil oxidants: from laboratory curiosity to clinical reality. J Immunol 202, 3127–3134. 31109945 10.4049/jimmunol.1900235

[81] Savina A , Jancic C , Hugues S , Guermonprez P , Vargas P , Moura IC , Lennon‐Duménil A‐M , Seabra MC , Raposo G & Amigorena S (2006) NOX2 controls phagosomal pH to regulate antigen processing during crosspresentation by dendritic cells. Cell 126, 205–218. 16839887 10.1016/j.cell.2006.05.035

[82] Mantegazza AR , Savina A , Vermeulen M , Perez L , Geffner J , Hermine O , Rosenzweig SD , Faure F & Amigorena S (2008) NADPH oxidase controls phagosomal pH and antigen cross‐presentation in human dendritic cells. Blood 112, 4712–4722. 18682599 10.1182/blood-2008-01-134791 PMC2597138

[83] Dingjan I , Paardekooper LM , Verboogen DRJ , von Mollard GF , Ter Beest M & van den Bogaart G (2017) VAMP8‐mediated NOX2 recruitment to endosomes is necessary for antigen release. Eur J Cell Biol 96, 705–714. 28688576 10.1016/j.ejcb.2017.06.007 PMC5641923

[84] Rybicka JM , Balce DR , Chaudhuri S , Allan ER & Yates RM (2012) Phagosomal proteolysis in dendritic cells is modulated by NADPH oxidase in a pH‐independent manner. EMBO J 31, 932–944. 22157818 10.1038/emboj.2011.440 PMC3280544

[85] Chen Y & Junger WG (2012) Measurement of oxidative burst in neutrophils. Methods Mol Biol 844, 115–124. 22262438 10.1007/978-1-61779-527-5_8 PMC4214271

[86] Ohkuma S & Poole B (1978) Fluorescence probe measurement of the intralysosomal pH in living cells and the perturbation of pH by various agents. Proc Natl Acad Sci USA 75, 3327–3331. 28524 10.1073/pnas.75.7.3327 PMC392768

[87] Geisow MJ , D'Arcy Hart P & Young MR (1981) Temporal changes of lysosome and phagosome pH during phagolysosome formation in macrophages: studies by fluorescence spectroscopy. J Cell Biol 89, 645–652. 6166620 10.1083/jcb.89.3.645 PMC2111800

[88] Canton J & Grinstein S (2017) Measuring phagosomal pH by fluorescence microscopy. Methods Mol Biol 1519, 185–199. 27815880 10.1007/978-1-4939-6581-6_12

[89] Savina A & Amigorena S (2007) Phagocytosis and antigen presentation in dendritic cells. Immunol Rev 219, 143–156. 17850487 10.1111/j.1600-065X.2007.00552.x

[90] Yates RM & Russell DG (2008) Real‐time spectrofluorometric assays for the lumenal environment of the maturing phagosome. Methods Mol Biol 445, 311–325. 18425459 10.1007/978-1-59745-157-4_20 PMC2759531

[91] Kim GH , Dayam RM , Prashar A , Terebiznik M & Botelho RJ (2014) PIKfyve inhibition interferes with phagosome and endosome maturation in macrophages. Traffic 15, 1143–1163. 25041080 10.1111/tra.12199

[92] Frost LS , Dhingra A , Reyes‐Reveles J & Boesze‐Battaglia K (2017) The use of DQ‐BSA to monitor the turnover of autophagy‐associated cargo. Methods Enzymol 587, 43–54. 28253971 10.1016/bs.mie.2016.09.052 PMC5338641

[93] Jancic C , Savina A , Wasmeier C , Tolmachova T , El‐Benna J , Dang PM , Pascolo S , Gougerot‐Pocidalo MA , Raposo G , Seabra MC *et al*. (2007) Rab27a regulates phagosomal pH and NADPH oxidase recruitment to dendritic cell phagosomes. Nat Cell Biol 9, 367–378. 17351642 10.1038/ncb1552

[94] Dayam RM , Saric A , Shilliday RE & Botelho RJ (2015) The phosphoinositide‐gated lysosomal Ca(2+) channel, TRPML1, is required for phagosome maturation. Traffic 16, 1010–1026. 26010303 10.1111/tra.12303

[95] Flannagan RS & Heinrichs DE (2018) A fluorescence based‐proliferation assay for the identification of replicating bacteria within host cells. Front Microbiol 9, 3084. 30619165 10.3389/fmicb.2018.03084 PMC6299164

[96] Mantegazza AR , Zajac AL , Twelvetrees A , Holzbaur EL , Amigorena S & Marks MS (2014) TLR‐dependent phagosome tubulation in dendritic cells promotes phagosome cross‐talk to optimize MHC‐II antigen presentation. Proc Natl Acad Sci USA 111, 15508–15513. 25313083 10.1073/pnas.1412998111 PMC4217451

[97] Nakamura N , Lill JR , Phung Q , Jiang Z , Bakalarski C , de Maziere A , Klumperman J , Schlatter M , Delamarre L & Mellman I (2014) Endosomes are specialized platforms for bacterial sensing and NOD2 signalling. Nature 509, 240–244. 24695226 10.1038/nature13133

[98] Mantegazza AR & Marks MS (2015) Visualizing toll‐like receptor‐dependent phagosomal dynamics in murine dendritic cells using live cell microscopy. Methods Mol Biol 1270, 191–203. 25702119 10.1007/978-1-4939-2309-0_15 PMC4356480

[99] Lu SM , Grinstein S & Fairn GD (2017) Quantitative live‐cell fluorescence microscopy during phagocytosis. Methods Mol Biol 1519, 79–91. 27815874 10.1007/978-1-4939-6581-6_6

[100] Swanson J , Bushnell A & Silverstein SC (1987) Tubular lysosome morphology and distribution within macrophages depend on the integrity of cytoplasmic microtubules. Proc Natl Acad Sci USA 84, 1921–1925. 3550801 10.1073/pnas.84.7.1921 PMC304553

[101] Robinson JM , Chiplonkar J & Luo Z (1996) A method for co‐localization of tubular lysosomes and microtubules in macrophages: fluorescence microscopy of individual cells. J Histochem Cytochem 44, 1109–1114. 8813075 10.1177/44.10.8813075

[102] McLean IW & Nakane PK (1974) Periodate‐lysine‐paraformaldehyde fixative. A new fixation for immunoelectron microscopy. J Histochem Cytochem 22, 1077–1083. 4374474 10.1177/22.12.1077

[103] Nunes P , Cornut D , Bochet V , Hasler U , Oh‐Hora M , Waldburger JM & Demaurex N (2012) STIM1 juxtaposes ER to phagosomes, generating Ca(2)(+) hotspots that boost phagocytosis. Curr Biol 22, 1990–1997. 23041196 10.1016/j.cub.2012.08.049

[104] Levin‐Konigsberg R & Grinstein S (2020) Phagosome‐endoplasmic reticulum contacts: kissing and not running. Traffic 21, 172–180. 31650670 10.1111/tra.12708

[105] Guermonprez P , Saveanu L , Kleijmeer M , Davoust J , Van Endert P & Amigorena S (2003) ER‐phagosome fusion defines an MHC class I cross‐presentation compartment in dendritic cells. Nature 425, 397–402. 14508489 10.1038/nature01911

[106] Blander JM & Medzhitov R (2004) Regulation of phagosome maturation by signals from toll‐like receptors. Science 304, 1014–1018. 15143282 10.1126/science.1096158

[107] Blander JM & Medzhitov R (2006) Toll‐dependent selection of microbial antigens for presentation by dendritic cells. Nature 440, 808–812. 16489357 10.1038/nature04596

[108] Mantegazza AR , Magalhaes JG , Amigorena S & Marks MS (2013) Presentation of phagocytosed antigens by MHC class I and II. Traffic 14, 135–152. 23127154 10.1111/tra.12026 PMC3538944

[109] Blander JM & Medzhitov R (2006) On regulation of phagosome maturation and antigen presentation. Nat Immunol 7, 1029–1035. 16985500 10.1038/ni1006-1029

[110] Napolitano G & Ballabio A (2016) TFEB at a glance. J Cell Sci 129, 2475–2481. 27252382 10.1242/jcs.146365 PMC4958300

[111] Martina JA & Puertollano R (2017) TFEB and TFE3: the art of multi‐tasking under stress conditions. Transcription 8, 48–54. 27892768 10.1080/21541264.2016.1264353 PMC5279710

[112] Visvikis O , Ihuegbu N , Labed SA , Luhachack LG , Alves AF , Wollenberg AC , Stuart LM , Stormo GD & Irazoqui JE (2014) Innate host defense requires TFEB‐mediated transcription of cytoprotective and antimicrobial genes. Immunity 40, 896–909. 24882217 10.1016/j.immuni.2014.05.002 PMC4104614

[113] Pastore N , Brady OA , Diab HI , Martina JA , Sun L , Huynh T , Lim JA , Zare H , Raben N , Ballabio A *et al*. (2016) TFEB and TFE3 cooperate in the regulation of the innate immune response in activated macrophages. Autophagy 12, 1240–1258. 27171064 10.1080/15548627.2016.1179405 PMC4968228

[114] Gray MA , Choy CH , Dayam RM , Ospina‐Escobar E , Somerville A , Xiao X , Ferguson SM & Botelho RJ (2016) Phagocytosis enhances lysosomal and bactericidal properties by activating the transcription factor TFEB. Curr Biol 26, 1955–1964. 27397893 10.1016/j.cub.2016.05.070 PMC5453720

[115] Steinman RM , Inaba K , Turley S , Pierre P & Mellman I (1999) Antigen capture, processing, and presentation by dendritic cells: recent cell biological studies. Hum Immunol 60, 562–567. 10426272 10.1016/s0198-8859(99)00030-0

[116] Hudson AW & Ploegh HL (2002) The cell biology of antigen presentation. Exp Cell Res 272, 1–7. 11740859 10.1006/excr.2001.5402

[117] Lennon‐Dumenil AM , Bakker AH , Wolf‐Bryant P , Ploegh HL & Lagaudriere‐Gesbert C (2002) A closer look at proteolysis and MHC‐class‐II‐restricted antigen presentation. Curr Opin Immunol 14, 15–21. 11790528 10.1016/s0952-7915(01)00293-x

[118] Mellman I (2005) Antigen processing and presentation by dendritic cells: cell biological mechanisms. Adv Exp Med Biol 560, 63–67. 15932021 10.1007/0-387-24180-9_9

[119] Villadangos JA , Schnorrer P & Wilson NS (2005) Control of MHC class II antigen presentation in dendritic cells: a balance between creative and destructive forces. Immunol Rev 207, 191–205. 16181337 10.1111/j.0105-2896.2005.00317.x

[120] Rocha N & Neefjes J (2008) MHC class II molecules on the move for successful antigen presentation. EMBO J 27, 1–5. 18046453 10.1038/sj.emboj.7601945 PMC2206127

[121] Zoncu R , Bar‐Peled L , Efeyan A , Wang S , Sancak Y & Sabatini DM (2011) mTORC1 senses lysosomal amino acids through an inside‐out mechanism that requires the vacuolar H(+)‐ATPase. Science 334, 678–683. 22053050 10.1126/science.1207056 PMC3211112

[122] Spanier JA , Frederick DR , Taylor JJ , Heffernan JR , Kotov DI , Martinov T , Osum KC , Ruggiero JL , Rust BJ , Landry SJ *et al*. (2016) Efficient generation of monoclonal antibodies against peptide in the context of MHCII using magnetic enrichment. Nat Commun 7, 11804. 27292946 10.1038/ncomms11804 PMC4909947

[123] Shimonkevitz R , Colon S , Kappler JW , Marrack P & Grey HM (1984) Antigen recognition by H‐2‐restricted T cells. II. A tryptic ovalbumin peptide that substitutes for processed antigen. J Immunol 133, 2067–2074. 6332146

[124] Rudensky A , Rath S , Preston‐Hurlburt P , Murphy DB & Janeway CA Jr (1991) On the complexity of self. Nature 353, 660–662. 1656278 10.1038/353660a0

[125] Barbey C , Pradervand E , Barbier N & Spertini F (2007) *Ex vivo* monitoring of antigen‐specific CD4+ T cells after recall immunization with tetanus toxoid. Clin Vaccine Immunol 14, 1108–1116. 17634505 10.1128/CVI.00004-07 PMC2043311

[126] Nair‐Gupta P , Baccarini A , Tung N , Seyffer F , Florey O , Huang Y , Banerjee M , Overholtzer M , Roche PA , Tampé R *et al*. (2014) TLR signals induce phagosomal MHC‐I delivery from the endosomal recycling compartment to allow cross‐presentation. Cell 158, 506–521. 25083866 10.1016/j.cell.2014.04.054 PMC4212008

[127] Shedlock DJ , Whitmire JK , Tan J , MacDonald AS , Ahmed R & Shen H (2003) Role of CD4 T cell help and costimulation in CD8 T cell responses during *Listeria monocytogenes* infection. J Immunol 170, 2053–2063. 12574376 10.4049/jimmunol.170.4.2053

[128] Tobar JA , Gonzalez PA & Kalergis AM (2004) Salmonella escape from antigen presentation can be overcome by targeting bacteria to Fc gamma receptors on dendritic cells. J Immunol 173, 4058–4065. 15356155 10.4049/jimmunol.173.6.4058

[129] McSorley SJ , Asch S , Costalonga M , Reinhardt RL & Jenkins MK (2002) Tracking salmonella‐specific CD4 T cells *in vivo* reveals a local mucosal response to a disseminated infection. Immunity 16, 365–377. 11911822 10.1016/s1074-7613(02)00289-3

[130] Cong Y , Feng T , Fujihashi K , Schoeb TR & Elson CO (2009) A dominant, coordinated T regulatory cell‐IgA response to the intestinal microbiota. Proc Natl Acad Sci USA 106, 19256–19261. 19889972 10.1073/pnas.0812681106 PMC2780781

[131] Sayes F , Blanc C , Ates LS , Deboosere N , Orgeur M Le , Chevalier F , Groschel MI , Frigui W , Song OR , Lo‐Man R *et al*. (2018) Multiplexed quantitation of intraphagocyte *Mycobacterium tuberculosis* secreted protein effectors. Cell Rep 23, 1072–1084. 29694886 10.1016/j.celrep.2018.03.125 PMC5946722

[132] Lim CY , Davis OB , Shin HR , Zhang J , Berdan CA , Jiang X , Counihan JL , Ory DS , Nomura DK & Zoncu R (2019) ER‐lysosome contacts enable cholesterol sensing by mTORC1 and drive aberrant growth signalling in Niemann‐Pick type C. Nat Cell Biol 21, 1206–1218. 31548609 10.1038/s41556-019-0391-5 PMC6936960

[133] Sepulveda FE , Maschalidi S , Colisson R , Heslop L , Ghirelli C , Sakka E , Lennon‐Dumenil AM , Amigorena S , Cabanie L & Manoury B (2009) Critical role for asparagine endopeptidase in endocytic toll‐like receptor signaling in dendritic cells. Immunity 31, 737–748. 19879164 10.1016/j.immuni.2009.09.013

[134] Langston HP , Ke Y , Gewirtz AT , Dombrowski KE & Kapp JA (2003) Secretion of IL‐2 and IFN‐gamma, but not IL‐4, by antigen‐specific T cells requires extracellular ATP. J Immunol 170, 2962–2970. 12626548 10.4049/jimmunol.170.6.2962

[135] Pfeifer JD , Wick MJ , Russell DG , Normark SJ & Harding CV (1992) Recombinant *Escherichia coli* express a defined, cytoplasmic epitope that is efficiently processed in macrophage phagolysosomes for class II MHC presentation to T lymphocytes. J Immunol 149, 2576–2584. 1383320

[136] Miller MA , Ganesan AP , Luckashenak N , Mendonca M & Eisenlohr LC (2015) Endogenous antigen processing drives the primary CD4+ T cell response to influenza. Nat Med 21, 1216–1222. 26413780 10.1038/nm.3958 PMC4629989

[137] Sanderson S & Shastri N (1994) LacZ inducible, antigen/MHC‐specific T cell hybrids. Int Immunol 6, 369–376. 8186188 10.1093/intimm/6.3.369

[138] Manz R , Assenmacher M , Pfluger E , Miltenyi S & Radbruch A (1995) Analysis and sorting of live cells according to secreted molecules, relocated to a cell‐surface affinity matrix. Proc Natl Acad Sci USA 92, 1921–1925. 7892200 10.1073/pnas.92.6.1921 PMC42394

[139] Gastelum‐Avina P , Lares‐Villa F , Espitia C , Valenzuela O , Robles‐Zepeda R , Velazquez C & Garibay‐Escobar A (2016) A rapid alternative method to evaluate T‐cell hybridoma activation using an improved cytokine (IL‐2) secretion assay. J Immunol Methods 438, 42–50. 27592266 10.1016/j.jim.2016.08.011

[140] Bevan MJ (1976) Cross‐priming for a secondary cytotoxic response to minor H antigens with H‐2 congenic cells which do not cross‐react in the cytotoxic assay. J Exp Med 143, 1283–1288. 1083422 10.1084/jem.143.5.1283 PMC2190184

[141] Joffre OP , Segura E , Savina A & Amigorena S (2012) Cross‐presentation by dendritic cells. Nat Rev Immunol 12, 557–569. 22790179 10.1038/nri3254

[142] Cruz FM , Colbert JD , Merino E , Kriegsman BA & Rock KL (2017) The biology and underlying mechanisms of cross‐presentation of exogenous antigens on MHC‐I molecules. Annu Rev Immunol 35, 149–176. 28125356 10.1146/annurev-immunol-041015-055254 PMC5508990

[143] Hildner K , Edelson BT , Purtha WE , Diamond M , Matsushita H , Kohyama M , Calderon B , Schraml BU , Unanue ER , Diamond MS *et al*. (2008) Batf3 deficiency reveals a critical role for CD8alpha+ dendritic cells in cytotoxic T cell immunity. Science 322, 1097–1100. 19008445 10.1126/science.1164206 PMC2756611

[144] Segura E , Albiston AL , Wicks IP , Chai SY & Villadangos JA (2009) Different cross‐presentation pathways in steady‐state and inflammatory dendritic cells. Proc Natl Acad Sci USA 106, 20377–20381. 19918052 10.1073/pnas.0910295106 PMC2787113

[145] Gros M & Amigorena S (2019) Regulation of antigen export to the cytosol during cross‐presentation. Front Immunol 10, 41. 30745902 10.3389/fimmu.2019.00041 PMC6360170

[146] Cebrian I , Visentin G , Blanchard N , Jouve M , Bobard A , Moita C , Enninga J , Moita LF , Amigorena S & Savina A (2011) Sec22b regulates phagosomal maturation and antigen crosspresentation by dendritic cells. Cell 147, 1355–1368. 22153078 10.1016/j.cell.2011.11.021

[147] Sengupta D , Graham M , Liu X & Cresswell P (2019) Proteasomal degradation within endocytic organelles mediates antigen cross‐presentation. EMBO J 38, e99266. 31271236 10.15252/embj.201899266 PMC6694219

[148] Touret N , Paroutis P , Terebiznik M , Harrison RE , Trombetta S , Pypaert M , Chow A , Jiang A , Shaw J , Yip C *et al*. (2005) Quantitative and dynamic assessment of the contribution of the ER to phagosome formation. Cell 123, 157–170. 16213220 10.1016/j.cell.2005.08.018

[149] Alloatti A , Kotsias F , Hoffmann E & Amigorena S (2016) Evaluation of cross‐presentation in bone marrow‐derived dendritic cells *in vitro* and splenic dendritic cells *ex vivo* using antigen‐coated beads. Bio Protoc 6, e2015. 10.21769/BioProtoc.2015 PMC5321431 28239619

[150] Clarke SR , Barnden M , Kurts C , Carbone FR , Miller JF & Heath WR (2000) Characterization of the ovalbumin‐specific TCR transgenic line OT‐I: MHC elements for positive and negative selection. Immunol Cell Biol 78, 110–117. 10762410 10.1046/j.1440-1711.2000.00889.x

[151] Faure F , Mantegazza A , Sadaka C , Sedlik C , Jotereau F & Amigorena S (2009) Long‐lasting cross‐presentation of tumor antigen in human DC. Eur J Immunol 39, 380–390. 19130478 10.1002/eji.200838669

[152] Segura E (2016) Cross‐presentation assay for human dendritic cells. Methods Mol Biol 1423, 189–198. 27142018 10.1007/978-1-4939-3606-9_14

[153] Shastri N & Gonzalez F (1993) Endogenous generation and presentation of the ovalbumin peptide/Kb complex to T cells. J Immunol 150, 2724–2736. 8454852

[154] Deckhut AM , Allan W , McMickle A , Eichelberger M , Blackman MA , Doherty PC & Woodland DL (1993) Prominent usage of V beta 8.3 T cells in the H‐2Db‐restricted response to an influenza A virus nucleoprotein epitope. J Immunol 151, 2658–2666. 7689611

[155] Valkenburg SA , Gras S , Guillonneau C , La Gruta NL , Thomas PG , Purcell AW , Rossjohn J , Doherty PC , Turner SJ & Kedzierska K (2010) Protective efficacy of cross‐reactive CD8+ T cells recognising mutant viral epitopes depends on peptide‐MHC‐I structural interactions and T cell activation threshold. PLoS Pathog 6, e1001039. 20711359 10.1371/journal.ppat.1001039 PMC2920842

[156] Mueller SN , Jones CM , Smith CM , Heath WR & Carbone FR (2002) Rapid cytotoxic T lymphocyte activation occurs in the draining lymph nodes after cutaneous herpes simplex virus infection as a result of early antigen presentation and not the presence of virus. J Exp Med 195, 651–656. 11877488 10.1084/jem.20012023 PMC2193766

[157] Udaka K , Tsomides TJ , Walden P , Fukusen N & Eisen HN (1993) A ubiquitous protein is the source of naturally occurring peptides that are recognized by a CD8+ T‐cell clone. Proc Natl Acad Sci USA 90, 11272–11276. 8248240 10.1073/pnas.90.23.11272 PMC47964

[158] Jiang Y , Lu Q , Wang Y , Xu E , Ho A , Singh P , Wang Y , Jiang Z , Yang F , Tietjen GT *et al*. (2020) Quantitating endosomal escape of a library of polymers for mRNA delivery. Nano Lett 20, 1117–1123. 32003222 10.1021/acs.nanolett.9b04426 PMC7195212

[159] Lu Q , Grotzke JE & Cresswell P (2018) A novel probe to assess cytosolic entry of exogenous proteins. Nat Commun 9, 3104. 30082832 10.1038/s41467-018-05556-z PMC6079096

[160] Latz E , Xiao TS & Stutz A (2013) Activation and regulation of the inflammasomes. Nat Rev Immunol 13, 397–411. 23702978 10.1038/nri3452 PMC3807999

[161] Klionsky DJ , Abdelmohsen K , Abe A , Abedin MJ , Abeliovich H , Acevedo Arozena A , Adachi H , Adams CM , Adams PD , Adeli K *et al*. (2016) Guidelines for the use and interpretation of assays for monitoring autophagy. Autophagy 12, 1–222. 26799652 10.1080/15548627.2015.1100356 PMC4835977

[162] Gross O (2012) Measuring the inflammasome. Methods Mol Biol 844, 199–222. 22262445 10.1007/978-1-61779-527-5_15

[163] Davis MJ & Swanson JA (2010) Technical advance: caspase‐1 activation and IL‐1beta release correlate with the degree of lysosome damage, as illustrated by a novel imaging method to quantify phagolysosome damage. J Leukoc Biol 88, 813–822. 20587739 10.1189/jlb.0310159 PMC2974426

[164] Joshi GN , Gilberti RM & Knecht DA (2017) Single cell analysis of phagocytosis, phagosome maturation, phagolysosomal leakage, and cell death following exposure of macrophages to silica particles. Methods Mol Biol 1519, 55–77. 27815873 10.1007/978-1-4939-6581-6_5

[165] Alvarez FJ , Herraez A & Tejedor MC (1996) Fluorescence analysis of carrier rat and human erythrocytes loaded with FITC‐dextran. Cytometry 24, 181–189. 8725668 10.1002/(SICI)1097-0320(19960601)24:2<181::AID-CYTO11>3.0.CO;2-N

[166] Pan DC , Myerson JW , Brenner JS , Patel PN , Anselmo AC , Mitragotri S & Muzykantov V (2018) Nanoparticle properties modulate their attachment and effect on carrier red blood cells. Sci Rep 8, 1615. 29371620 10.1038/s41598-018-19897-8 PMC5785499

[167] Jia J , Claude‐Taupin A , Gu Y , Choi SW , Peters R , Bissa B , Mudd MH , Allers L , Pallikkuth S , Lidke KA *et al*. (2020) Galectin‐3 coordinates a cellular system for lysosomal repair and removal. Dev Cell 52, 69–87 e8. 31813797 10.1016/j.devcel.2019.10.025 PMC6997950

[168] Bastiat‐Sempe B , Love JF , Lomayesva N & Wessels MR (2014) Streptolysin O and NAD‐glycohydrolase prevent phagolysosome acidification and promote group A Streptococcus survival in macrophages. MBio 5, e01690‐14. 25227466 10.1128/mBio.01690-14 PMC4172074

[169] Meunier E , Dick MS , Dreier RF , Schurmann N , Kenzelmann Broz D , Warming S , Roose‐Girma M , Bumann D , Kayagaki N , Takeda K *et al*. (2014) Caspase‐11 activation requires lysis of pathogen‐containing vacuoles by IFN‐induced GTPases. Nature 509, 366–370. 24739961 10.1038/nature13157

[170] Ray K , Bobard A , Danckaert A , Paz‐Haftel I , Clair C , Ehsani S , Tang C , Sansonetti P , Tran GV & Enninga J (2010) Tracking the dynamic interplay between bacterial and host factors during pathogen‐induced vacuole rupture in real time. Cell Microbiol 12, 545–556. 20070313 10.1111/j.1462-5822.2010.01428.x

[171] Vivar OI , Magalhaes JG & Amigorena S (2016) Measurement of export to the cytosol in dendritic cells using a cytofluorimetry‐based assay. Methods Mol Biol 1423, 183–188. 27142017 10.1007/978-1-4939-3606-9_13

[172] Meunier E & Broz P (2015) Quantification of cytosolic vs. vacuolar Salmonella in primary macrophages by differential permeabilization. J VisExp 101, e52960. 10.3791/52960 PMC4545148 26274778

[173] Yu L , McPhee CK , Zheng L , Mardones GA , Rong Y , Peng J , Mi N , Zhao Y , Liu Z , Wan F *et al*. (2010) Termination of autophagy and reformation of lysosomes regulated by mTOR. Nature 465, 942–946. 20526321 10.1038/nature09076 PMC2920749

[174] Freeman SA & Grinstein S (2018) Resolution of macropinosomes, phagosomes and autolysosomes: osmotically driven shrinkage enables tubulation and vesiculation. Traffic 19, 965–974. 30159984 10.1111/tra.12614

[175] Fazeli G , Stetter M , Lisack JN & Wehman AM (2018) *C. elegans* blastomeres clear the corpse of the second polar body by LC3‐associated phagocytosis. Cell Rep 23, 2070–2082. 29768205 10.1016/j.celrep.2018.04.043

[176] Champion JA , Walker A & Mitragotri S (2008) Role of particle size in phagocytosis of polymeric microspheres. Pharm Res 25, 1815–1821. 18373181 10.1007/s11095-008-9562-y PMC2793372

[177] Haney MS , Bohlen CJ , Morgens DW , Ousey JA , Barkal AA , Tsui CK , Ego BK , Levin R , Kamber RA , Collins H *et al*. (2018) Identification of phagocytosis regulators using magnetic genome‐wide CRISPR screens. Nat Genet 50, 1716–1727. 30397336 10.1038/s41588-018-0254-1 PMC6719718

[178] Montano F , Grinstein S & Levin R (2018) Quantitative phagocytosis assays in primary and cultured macrophages. Methods Mol Biol 1784, 151–163. 29761397 10.1007/978-1-4939-7837-3_15

[179] Zent CS & Elliott MR (2017) Maxed out macs: physiologic cell clearance as a function of macrophage phagocytic capacity. FEBS J 284, 1021–1039. 27863012 10.1111/febs.13961 PMC5378628

[180] Wang Y , Subramanian M , Yurdagul A Jr , Barbosa‐Lorenzi VC , Cai B , de Juan‐Sanz J , Ryan TA , Nomura M , Maxfield FR & Tabas I (2017) Mitochondrial fission promotes the continued clearance of apoptotic cells by macrophages. Cell 171, 331–345 e22. 28942921 10.1016/j.cell.2017.08.041 PMC5679712

[181] Pope C , Kim SK , Marzo A , Masopust D , Williams K , Jiang J , Shen H & Lefrancois L (2001) Organ‐specific regulation of the CD8 T cell response to *Listeria monocytogenes* infection. J Immunol 166, 3402–3409. 11207297 10.4049/jimmunol.166.5.3402

[182] Underhill DM (2003) Macrophage recognition of zymosan particles. J Endotoxin Res 9, 176–180. 12831459 10.1179/096805103125001586

[183] Kissing S , Hermsen C , Repnik U , Nesset C , von Bargen K , Griffiths G , Ichihara A , Lee BS , Schwake M & De Brabander J (2015) Vacuolar ATPase in phagosome‐lysosome fusion. J Biol Chem 290, 14166–14180. 25903133 10.1074/jbc.M114.628891 PMC4447986

[184] Frost LS , Lopes VS , Bragin A , Reyes‐Reveles J , Brancato J , Cohen A , Mitchell CH , Williams DS & Boesze‐Battaglia K (2015) The contribution of melanoregulin to microtubule‐associated protein 1 Light Chain 3 (LC3) associated phagocytosis in retinal pigment epithelium. Mol Neurobiol 52, 1135–1151. 25301234 10.1007/s12035-014-8920-5 PMC5531606

[185] Prashar A , Khan SI & Terebiznik MR (2017) Filamentous bacteria as targets to study phagocytosis. Methods Mol Biol 1519, 311–323. 27815889 10.1007/978-1-4939-6581-6_21

[186] Lindner B , Burkard T & Schuler M (2020) Phagocytosis assays with different pH‐sensitive fluorescent particles and various readouts. Biotechniques 68, 245–250. 32079414 10.2144/btn-2020-0003

[187] Levin R , Hammond GR , Balla T , De Camilli P , Fairn GD & Grinstein S (2017) Multiphasic dynamics of phosphatidylinositol 4‐phosphate during phagocytosis. Mol Biol Cell 28, 128–140. 28035045 10.1091/mbc.E16-06-0451 PMC5221617

[188] Schlam D , Bohdanowicz M , Chatgilialoglu A , Steinberg BE , Ueyama T , Du G , Grinstein S & Fairn GD (2013) Diacylglycerol kinases terminate diacylglycerol signaling during the respiratory burst leading to heterogeneous phagosomal NADPH oxidase activation. J Biol Chem 288, 23090–23104. 23814057 10.1074/jbc.M113.457606 PMC3743482

[189] VanderVen BC , Yates RM & Russell DG (2009) Intraphagosomal measurement of the magnitude and duration of the oxidative burst. Traffic 10, 372–378. 19183302 10.1111/j.1600-0854.2009.00877.x PMC2736473

[190] Cuellar‐Mata P , Jabado N , Liu J , Furuya W , Finlay BB , Gros P & Grinstein S (2002) Nramp1 modifies the fusion of Salmonella typhimurium‐containing vacuoles with cellular endomembranes in macrophages. J Biol Chem 277, 2258–2265. 11700301 10.1074/jbc.M105508200

[191] Nayak JV , Hokey DA , Larregina A , He Y , Salter RD , Watkins SC & Falo LD Jr (2006) Phagocytosis induces lysosome remodeling and regulated presentation of particulate antigens by activated dendritic cells. J Immunol 177, 8493–8503. 17142747 10.4049/jimmunol.177.12.8493

[192] Binker MG , Cosen‐Binker LI , Terebiznik MR , Mallo GV , McCaw SE , Eskelinen EL , Willenborg M , Brumell JH , Saftig P , Grinstein S *et al*. (2007) Arrested maturation of Neisseria‐containing phagosomes in the absence of the lysosome‐associated membrane proteins, LAMP‐1 and LAMP‐2. Cell Microbiol 9, 2153–2166. 17506821 10.1111/j.1462-5822.2007.00946.x

[193] Johnson DE , Ostrowski P , Jaumouille V & Grinstein S (2016) The position of lysosomes within the cell determines their luminal pH. J Cell Biol 212, 677–692. 26975849 10.1083/jcb.201507112 PMC4792074

[194] Rabinowitz S , Horstmann H , Gordon S & Griffiths G (1992) Immunocytochemical characterization of the endocytic and phagolysosomal compartments in peritoneal macrophages. J Cell Biol 116, 95–112. 1730752 10.1083/jcb.116.1.95 PMC2289258

[195] Sasai M , Linehan MM & Iwasaki A (2010) Bifurcation of Toll‐like receptor 9 signaling by adaptor protein 3. Science 329, 1530–1534. 20847273 10.1126/science.1187029 PMC3063333

